# Independent and Complementary Functions of Caf1b and Hir1 for Chromatin Assembly in *Tetrahymena thermophila*

**DOI:** 10.3390/cells12242828

**Published:** 2023-12-13

**Authors:** Huijuan Hao, Chenhui Ren, Yinjie Lian, Min Zhao, Tao Bo, Jing Xu, Wei Wang

**Affiliations:** 1 Key Laboratory of Chemical Biology and Molecular Engineering of Ministry of Education, Institute of Biotechnology, Shanxi University, Taiyuan 030006, China; 201813002002@email.sxu.edu.cn (H.H.); rch17899@163.com (C.R.); 20230510@hbmu.edu.cn (Y.L.); 202123001025@email.sxu.edu.cn (M.Z.); botao@sxu.edu.cn (T.B.); 2 School of Life Science, Shanxi University, Taiyuan 030006, China; 3 Shanxi Key Laboratory of Biotechnology, Taiyuan 030006, China

**Keywords:** *Tetrahymena thermophila*, histone chaperone, Caf1b, Hir1, chromatin assembly

## Abstract

Histones and DNA associate to form the nucleosomes of eukaryotic chromatin. Chromatin assembly factor 1 (CAF-1) complex and histone regulatory protein A (HIRA) complex mediate replication-couple (RC) and replication-independent (RI) nucleosome assembly, respectively. CHAF1B and HIRA share a similar domain but play different roles in nucleosome assembly by binding to the different interactors. At present, there is limited understanding for the similarities and differences in their respective functions. *Tetrahymena thermophila* contains transcriptionally active polyploid macronuclei (MAC) and transcriptionally silent diploid micronuclei (MIC). Here, the distribution patterns of Caf1b and Hir1 exhibited both similarities and distinctions. Both proteins localized to the MAC and MIC during growth, and to the MIC during conjugation. However, Hir1 exhibited additional signaling on parental MAC and new MAC during sexual reproduction and displayed a punctate signal on developing anlagen. Caf1b and Hir1 only co-localized in the MIC with Pcna1 during conjugation. Knockdown of *CAF1B* impeded cellular growth and arrested sexual reproductive development. Loss of *HIR1* led to MIC chromosome defects and aborted sexual development. Co-interference of *CAF1B* and *HIR1* led to a more severe phenotype. Moreover, *CAF1B* knockdown led to the up-regulation of *HIR1* expression, while knockdown of *HIR1* also led to an increase in *CAF1B* expression. Furthermore, Caf1b and Hir1 interacted with different interactors. These results showed that CAF-1 and Hir1 have independent and complementary functions for chromatin assembly in *T. thermophila*.

## 1. Introduction

In eukaryotic cells, chromatin is composed of repetitive nucleosomes. The ~147 bp DNA is tightly wrapped around the histone core octamer consisting of H3, H4, H2A, and H2B subunits to form nucleosomes and is further stabilized by the H1 linker histone [1]. The canonical histones and histone variants play a pivotal role in precisely modulating chromatin dynamics and regulating accessibility to specific DNA regions [2]. The nucleosome conveys specific genetic information through covalently modified or incorporated histone variants. The assembly, disassembly, and reassembly of nucleosomes are precisely orchestrated processes that underlie crucial cellular functions such as DNA replication, transcription, and repair [3,4]. Different histones and their variants exhibit various chromatin assembly pathways. Classic histone H3.1 is deposited onto chromatin via the replication-couple (RC) nucleosome assembly pathway facilitated by chromatin assembly factor 1 (CAF-1) [5,6]. In contrast to H3.1, the variant of histone H3 known as H3.3 follows an alternative pathway facilitated by HIRA for its incorporation into the nucleosome gap [5,7]. Recently, based on BioID proximity labeling, CAF-1 not only deposits the H3.1 histone variant during the S phase but also associates with H3.3 throughout the cell cycle in mammalian cells [8]. The centromere histone A (CENP-A) is deposited on the centromere with the assistance of the chaperone protein HJURP (holiday junction recognition protein) [9].

CAF-1 is ubiquitously present in all eukaryotic cells [10,11]. It represents an evolutionarily conserved histone chaperone complex essential for various fundamental processes in eukaryotes, including chromatin repair following DNA synthesis, cell cycle progression, as well as the establishment and maintenance of heterochromatin [12,13,14]. In most eukaryotes, CAF-1 is a heterotrimeric complex comprising the large subunit p150/CHAF1A, the medium subunit p60/CHAF1B, and the small subunit p48/CHAF1C [15,16,17]. The CHAF1B contains seven WD40 repeats and a B-domain-like motif that binds to Asf1 [18,19]. In vertebrate cells, the deletion of any one of the subunits in CAF-1 causes a delay in the S phase, decelerated DNA synthesis, impaired assembly of replication-coupled nucleosomes, and ultimately cell death [20]. Conversely, the removal of the subunit in CAF-1 in yeast does not affect cell survival [21,22]. In budding yeast, CAF-1 interacts with Cse4 and colocalizes with the Cse4 at the centromeres in the absence of Scm3 [23]. Overexpressed CENP-A was mislocated in CHAF1B-depleted HeLa cells [24]. CHAF1B deletion leads to abnormal hematopoietic reconstitution in bone marrow cells [25].

In mammals, the HIR complex is a heterotrimer consisting of HIRA, ubinuclein1/2 (Ubn1/2), and calcineurin-binding protein 1 (Cabin1), which shares homology with the complexes formed by hir1, hir2, hpc2, and hir3 in yeast [7,26,27]. The HIRA comprises three highly conserved domains: the N-terminal WD40 domain, B domain, and the C-terminal Hira domain [28,29]. The WD40 domain interacts with Ubn1/2 binding to H3.3; the B domain interacts with Asf1; and the C-terminal Hira domain interacts with Cabin1 [30,31,32]. The knockdown of Hira leads to concurrent depletion of Cabin1 and Ubn1 proteins [5]. Mutations in HIRA in mice result in defective gastric development and abnormal patterning [33]. Removing Hira from the hematopoietic system induces rapid loss of hematopoietic stem cells [34]. In mouse oogenesis, absence of Hira causes severe chromatin defects, maturation impairments, and accumulation of damaged DNA [35].

*Tetrahymena thermophila* is a ciliate protozoan exhibiting nuclear dimorphism, characterized by the transcriptionally active polyploid macronucleus MAC and the transcriptionally silent diploid germline MIC. During vegetative growth, MACs undergo amitotic division, while MICs divide through mitosis [36]. During conjugation development, the MIC undergoes meiosis, followed by mitosis of pronuclei and two subsequent mitotic divisions of the postzygotic nucleus. Subsequently, the new MAC anlagen undergoes endoreplication and internal eliminated sequences (IES) deletion, while the parental MAC gradually degrades. Finally, the paired cells separate and form an exconjugant containing two MACs and one MIC. In the nutrient-sufficient environment, the exconjugant restarts proliferation [37]. In *Tetrahymena,* there are five variants of histone H3, namely *HHT1-4* and *CNA1* (CENP-A-like 1). *HHT1* and *HHT2* encode for the canonical H3 known as H3.1, *HHT3* and *HHT4* encode similar H3 variants, H3.3 and H3.4. The H3.1 is deposited onto chromatin by a DNA RC pathway; in contrast, H3.3 is deposited mainly through the RI pathway and weakly through an RC pathway [38]. The histones exhibit variations in their composition and post-translational modifications. In most eukaryotes, H3.1 and H3.3 differ by only four or five amino acids while *Tetrahymena* H3.1 and H3.3 differ at 16 residues [38,39]. Furthermore, there are slow-migrating H3^S^ and fast-migrating H3^F^ unique to MIC in *Tetrahymena*. H3^F^ is generated by splitting the six amino acids at the N-terminal of H3 [40]. Hence, trimethylation of H3K4 is only available in MAC. Currently, there remains a limited understanding regarding the assembly of histones with different sizes and variants into DNA, as well as the interplay between RC nucleosome assembly and RI nucleosome assembly in *T. thermophila*. Asf1 and Nrp1 represent pivotal upstream core proteins involved in nucleosome assembly. A decrease in Nrp1 leads to abnormal MIC mitosis and MAC amitosis [41,42]. In this study, we found that Caf1b localized in both MACs and MICs during the vegetative growth stage; then, it localized in the MIC and replicated anlagen during sexual development. In contrast, Hir1 exhibited localization in both MAC and MIC throughout vegetative growth, starvation, and sexual reproduction stages. Loss of *CAF1B* affected cellular proliferation during asexual reproduction and led to abnormal meiosis during the sexual development stage. Impaired expression of *HIR1* compromises the integrity of MIC chromatin. The co-interference of *CAF1B* and *HIR1* led to a more serious phenotype than single-gene knockdown mutants. CAF-1 and Hir1 exhibit independent and complementary functions for chromatin assembly in *Tetrahymena*.

## 2. Materials and Methods

### 2.1. Strains Culture and Mating

*T. thermophila* wild-type cell lines B2086 (II), CU428 (VII), and CU427 (VI) were obtained from the National Tetrahymena Stock Center (http://tetrahymena.vet.cornell.edu/, initially accessed on 10 September 2008, currently is stored in our Lab). The cells were grown in Super Proteose Peptone (SPP) medium (0.2% glucose, 0.1% yeast extract, 1% proteose peptone, and 0.003% EDTA-Fe) at 30 °C. Log-phase cells were washed with 10 mmol/L Tris-HCl (pH 7.4) and resuspended in the same buffer for 18–24 h. Equal numbers of cells from two different mating types were mixed and induced mating.

### 2.2. Identification of Caf1b and Hir1

The sequences of *CAF1B* (TTHERM_00219420) and *HIR1* (TTHERM_00046490) were retrieved using BLAST search from the Tetrahymena Genome Database (http://www.ciliate.org, accessed on 9 June 2021). Protein clustering analysis was performed using MEGA11.0.11, while IBS 1.0.3 was utilized for mapping the domains of these proteins.

### 2.3. Phylogenetic Tree Construction

The homologous proteins of Caf1b and Hir1 were searched through sequence alignment in NCBI (https://www.ncbi.nlm.nih.gov/, accessed on 18 May 2022) or UniProt (https://www.uniprot.org/, accessed on 20 May 2022) databases, and their amino acid sequences were downloaded. The sequences of WD domains of Caf1b and Hir1 were retrieved in the Conserved Domain Database (https://www.ncbi.nlm.nih.gov/Structure/cdd/wrpsb.cgi, accessed on 5 June 2022). Next, these amino acid sequences were subjected to ClustalW-based sequence alignment in MEGA11.0.11 to eliminate non-specific regions. Finally, phylogenetic trees were constructed using the neighbor-joining method.

### 2.4. Construct of HA-Tagged Caf1b and Hir1

The C-terminal region (639 bp) and 3’ flanking sequence (501 bp) of the *CAF1B* were amplified using *CAF1B*-HA-5F/5R and *CAF1B*-HA-3F/3R (Table S1). Meanwhile, the C-terminal region (635 bp) and 3’ flanking sequence (524 bp) of the *HIR1* were amplified using *HIR1*-HA-5F/5R and *HIR1*-HA-3F/3R (Table S1). Subsequently, the amplified fragments were ligated to the pMD-19T vector. The C-terminal fragment was then digested with *Sac* I and *Not* I, followed by ligation into the pNeo4 vector. Similarly, the flanking sequence was digested with *Xho* I and *Kpn* I before being ligated to pHA-Neo4 using DNA ligase. Enriched fragments obtained through primer Shoot-*CAF1B*-HA-F/R and Shoot-*HIR1*-HA-F/R were introduced into *T. thermophila* cells via biolistic bombardment (SCIENTZ, Ningbo, China). The transformed cells were subjected to varying concentrations of paromomycin, from low to high, for better substitution. The mutants were identified by PCR using the primer Shoot-*CAF1B*-HA-F/R and Shoot-*HIR1*-HA-F/R.

### 2.5. Knockout of CAF1B and HIR1

The 5’ (947 bp) and 3’ (506 bp) flanking sequences of *CAF1B* were amplified using K-*CAF1B*-5F/5R and K-*CAF1B*-3F/3R primers, followed by ligation with pMD-19T. At the same time, the 5’ (454 bp) and 3’ (590 bp) flanking sequences of the *HIR1* were amplified using K-*HIR1*-5F/5R and K-*HIR1*-3F/3R primers, followed by ligation with pMD-19T. Subsequently, the fragments of 5’ flanking sequences cleaved by *Sac* I/*Not* I and the fragments of 3’ flanking sequences cleaved by *Xho* I/*Kpn* I were purified and ligated to the pNeo4 vector. The resulting recombinant plasmids were digested with *Sac* I and *Kpn* I and transferred to *T. thermophila* via biolistic bombardment (SCIENTZ, Ningbo, China). Transformants exhibiting paromomycin resistance were selected. The mutants were identified by PCR using the primer J-*CAF1B*-F/R and J-*HIR1*-F/R.

### 2.6. Construction of Conditionally Induced Interference Mutants

The conserved fragment (516 bp) from *CAF1B* were amplified using RNAi-*CAF1B*-1F/1R and RNAi-*CAF1B*-2F/2R, which only differed in the enzymatic cleavage site. Similarly, we amplified the conserved fragment (594 bp) of *HIR1* were amplified with RNAi-*HIR1*-1F/1R and RNAi-*HIR1*-2F/2R primers. The amplified fragments were ligated to pMD-19T. Fragments digested by *Pst* I/*Sma* I and *Bam*H I/*Pme* I were ligated with the phpNeo5 vector cleaved using the same enzyme. Plasmids that successfully formed hairpin structures were then digested with *Xho* I/*Not* I and transformed into *T. thermophila* using biolistic bombardment (SCIENTZ, Ningbo, China). Transformants were selected in the presence of paromomycin, and interference efficiency was assessed by qRT-PCR using RT-*CAF1B*-F/R or RT-*HIR1*-F/R.

### 2.7. RT-PCR and qPCR

The cultured cells were subjected to RNA isolation using Trizol (Yeasen Biotechnology, Shanghai, China). Subsequently, 600 ng of total RNA with the gDNA removed was reverse-transcribed into cDNA using a kit (Yeasen Biotechnology, Shanghai, China). The expression levels of target genes were quantified using the primers listed in Table S1. SYBR Green Master Mix (Yeasen Biotechnology, Shanghai, China) and Bio-Rad CFX Connect were employed for gene expression analysis. The internal reference used was 17SrRNA.

### 2.8. Indirect Immunofluorescence Staining

Log-phase cells (5 mL) were fixed with 20 μL Schaudinn’s fixative (saturated HgCl_2_/ ethanol, 2:1). The fixed cells were uniformly distributed on poly-L-lysine-coated coverslips and subsequently hydrated with PBST (0.05% Triton X-100) for 10 min. Then, the cells were blocked in a solution containing 3% BSA, 10% normal goat serum, and 0.05% Triton X-100 in PBS for one hour. Subsequently, the cells were incubated with rabbit anti-HA (dilution ratio of 1:500, #3724S, CST, Danvers, MA, USA), mouse anti-HA (dilution ratio of 1:200, #2367S, CST, Danvers, MA, USA), and rabbit anti-Pcna1 (dilution ratio of 1:500, WG11849, ABclonal, Wuhan, China) antibodies at 4 °C overnight. Then, the samples were washed three times using PBST and then incubated for an hour at room temperature with FITC-conjugated anti-rabbit IgG antibody (dilution ratio of 1:1000, AQ132F, Millipore, Billerica, MA, USA) or anti-mouse IgG antibody (dilution ratio of 1:500, AP192R, Millipore, Billerica, MA, USA). After washing again with PBST, the samples were stained with 1 μg/mL DAPI for 15 min. Finally, the samples were observed using a Delta Vision Elite deconvolution microscope system (Applied Precision/GE Healthcare, Boston, Massachusetts, USA) or Olympus Microscope BX51 (OLYMPUS, Tokyo, Japan).

### 2.9. Western Blotting

Cells (1 × 10^7^) were collected in 100 μL PBS containing cocktails and then sonicated for disruption. The supernatant was collected and subsequently boiled for 5 min with the addition of 5 × loading buffer. Equal amounts of cell lysates were separated by SDS-PAGE, subsequently transferred onto PVDF membranes, and then blocked with 5% milk. Following blocking, the membranes were incubated overnight at 4 °C with antibodies against HA (dilution: 1:500, #3724S, CST, Danvers, MA, USA), His (dilution: 1:500, D191001, BBI, Shanghai, China), and GST (dilution: 1:500, D190101, BBI, Shanghai, China). The samples were washed and incubated with HRP-conjugated secondary antibody (dilution: 1:1000, A9169, Sigma, Santa Clara, CA, USA) or (dilution: 1:1000, A4416, Sigma, Santa Clara, CA, USA) for 1 h at room temperature. After cleaning and adding ECL luminescent solution, the visualization was obtained using X-ray film or GelView 6000Pro II (BLT, Guangzhou, China).

### 2.10. Micronuclear Integrity Assay

The genomes from wild-type and *HIR1*KD mutants were extracted. Ten pairs of primers of micronucleus-specific chromosome breakage sequences (Cbs) were used to identify the integrity of MIC by PCR (Table S1). The PCR cycling conditions consisted of an initial denaturation step at 94 °C for 5 min, followed by 32 cycles of denaturation at 94 °C for 30 s, annealing at 56 °C for 30 s, extension at 68 °C for 60 s, and a final extension step at 68 °C for 5 min.

### 2.11. Co-Immunoprecipitation and Mass Spectrometry

Cells (1 × 10^7^) were collected and resuspended in 500 μL PBS containing 100 × Inhibitor Cocktail (Thermo Fisher Scientific, Waltham, MA, USA) and 0.5 M EDTA. Following ultrasonic disruption and subsequent centrifugation, the resulting supernatant was incubated with 20 μL of packed anti-HA agarose beads (Thermo Fisher Scientific, Waltham, MA, USA) in a spin column overnight at 4 °C with continuous end-to-end mixing. The samples were then subjected to short centrifugation for 10 s followed by seven washes using TBST buffer (25 mmol/L Tris-HCl, 0.15 mol/L NaCl, pH 7.2, 0.05% Tween-20). After each wash step, the samples were briefly centrifuged again. Subsequently, the HA-tagged protein was eluted from the beads using 25 μL non-reducing sample buffer and then boiled for 5 min. An aliquot of 5.5 μL was used for Western blot analysis while 6.5 μL was used for silver staining; any remaining sample was utilized for mass spectrometry analysis (Shanghai Applied Protein Technology Shanghai, China). After reduction and alkylation, Trypsin was added to the sample (mass ratio 1:50) and enzymolized at 37 °C for 20 h. After desalting, the enzymolysis product was lyophilized, redissolved in 0.1% FA solution, and stored at −20 °C for further use. After the column was balanced with 95% liquid A (0.1% formic acid aqueous solution), the sample was loaded into the Trap column using an automatic sampler. A total of 20 fragments were collected after each full scan (MS2 Scan). The identified protein results were obtained. We denoted the ratio of Caf1b-HA or Hir1-HA to the number of WT peptides as foldchange and proteins with a ratio more than 4 were identified as interactors of Caf1b or Hir1.

### 2.12. Pull-Down Assay

The codon-optimized fragments of B-like 1–3 from Caf1b and B domain of Hir1 were ligated into the pGEX-6P-1, while Asf1 was ligated into the pESUMO vector. Subsequently, the recombinant plasmids were transformed into *E. coli* BL21 (DE3) and induced with 0.1 mM IPTG for expression at 37 °C or 16 °C. After sonication, the supernatant of GST, GST-B-like 1–3, and GST-B were added to chromatography columns with GST-sefinose resin (BBI Life Sciences, Shanghai, China) and incubated in a shaker at 4 °C for 5 h after the addition of an equal amount of the trapped protein Asf1. Following this step, centrifugation at 1500 rpm for 1 min was performed. The gel beads were washed with PBST. Finally, proteins were eluted using 100 μL of PBS supplemented with 10 mM GSH. The identification of bait proteins and trapped proteins was analyzed using Western blot.

## 3. Results

### 3.1. Characterization of Caf1b and Hir1

**Figure 1:**
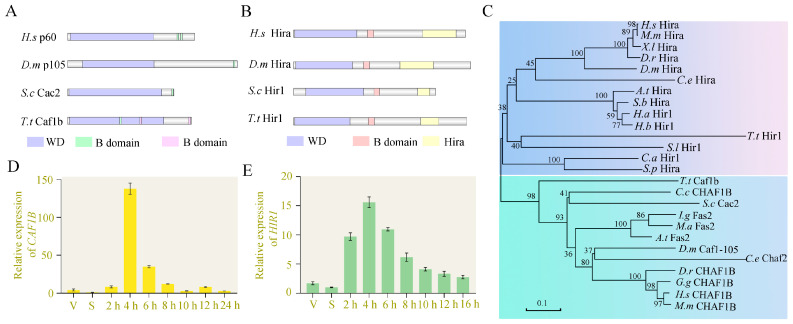
The characterization of Caf1b and Hir1. (**A**) Schematic diagram of the domain of Caf1b. The domain of Caf1b was delineated based on the conserved domain identified by NCBI. The length of the silver rectangles indicates the corresponding amino acid lengths. Purple rectangles denote WD domains, green rectangles represent conserved B-domain-like motifs, and pink rectangles indicate not conserved B-domain-like motifs. (**B**) Schematic diagram of Hir1 domain. The length of the silver rectangles indicates the corresponding amino acid lengths. B domain, a motif that binds to Asf1, is visually represented by red rectangles. Hira, a domain-specific to Hir1, and its analogous proteins are denoted by yellow rectangles. WD domains are marked with purple rectangles. (**C**) Phylogenetic tree of WD from Caf1b, Hir1, and its homologs in different species. The amino acid sequences were aligned by ClustalW to construct a neighbor-joining tree. The upper rectangle indicates the homologous protein of Hir1 and the lower rectangle indicates the homologous protein of Caf1b. *H.s* Hira (*Homo sapiens*, NP_003316.3); *M.m* Hira (*Mus musculus*, XP_006521863.1); *X.l* Hira (*Xenopus laevis*, XP_018099608.1); *D.r* Hira (*Danio rerio*, XP_696478.2); *D.m* Hira (*Drosophila melanogaster*, NP_572401.2); *C.e* Hira (*Caenorhabditis elegans*, NP_498101.2); *A.t* Hira (*Arabidopsis thaliana*, NP_001319681.1); *S.b* Hira (*Sorghum bicolor*, XP_002463803.1); *H.a* Hira (*Helianthus annuus*, XP_022000519.1); *H.b* Hira (*Hevea brasiliensis*, XP_021665743.2); *T.t* Hir1 (*Tetrahymena thermophila*, XP_001014644.1); *S.l* Hira (*Stylonychia lemnae*, CDW71962.1); *C.a* Hir1 (*Candida albicans*, XP_019330737.1); *S.p* Hira (*Schizosaccharomyces pombe*, NP_596575.1); *T.t* Caf1b (*Tetrahymena thermophila*, XP_001020614.1); *C.c* CHAF1B (*Choanephora cucurbitarum*, OBZ88111.1); *S.c* Cac2 (*Saccharomyces cerevisiae*, Q04199.1); *I.g* Fas2 (*Impatiens glandulifera*, XP_047327461.1); *M.a* Fas2 (*Mercurialis annua*, XP_050204251.1); *A.t* Fas2 (*Arabidopsis thaliana*, Q9SXY1.1); *D,m* Caf1-105 (*Drosophila melanogaster*, NP_610589.2); *C.e* Chaf-2 (*Caenorhabditis elegans*, NP_490901.2); *D.r* CHAF1B (*Danio rerio*, NP_001315058.1); *G.g* CHAF1B (*Gallus gallus*, Q5R1S9); *H.s* CHAF1B (*Homo sapiens*, Q13112); *M.m* CHAF1B *(Mus musculus*, Q9D0N7). (**D**) Expression profile of *CAF1B*. The gene fragments were amplified from wild-type cells using qRT-PCR during the vegetative growth (V), starvation (S), and sexual development (2, 4, 6, 8, 10, 12, 24 h) stages. (**E**) Expression profile of *HIR1* at the vegetative growth (V), starvation (S), and sexual development (2, 4, 6, 8, 10, 12, 16 h) stages.

CAF-1 and HIRA complexes mediate RC and RI histone deposition, respectively [7,43]. *CAF1B* (TTHERM_00219420) and *HIR1* (TTHERM_00046490) are homologs of p60 and HIRA in *T. thermophila*. Caf1b was predicted to contain the WD domain, a conserved B-domain-like motif, and two non-conservative B-domain-like motifs (Figure 1A). The sequence from the N-terminal to C-terminal was designated as B-like 1 to 3 (Supplementary Figure S1A). Hir1 comprises a WD domain, a B domain motif, and a Hira domain (Figure 1B). The B domain of Hir1 is conserved across species (Supplementary Figure S1B). The WD domain exhibits a characteristic seven-bladed β-propeller structure. WD domains serve as crucial subunits within multiprotein complexes involved in diverse signaling pathways [44]. The phylogenetic analysis of the WD domain from Caf1b and Hir1 indicated their evolutionary conservation and divergence between these two protein families (Figure 1C). The expression of *CAF1B* and *HIR1* is low during growth and starvation but reaches its peak at 4–6 h of sexual reproduction (Figure 1D,E).

### 3.2. Dynamic Localization of Caf1b and Hir1

**Figure 2:**
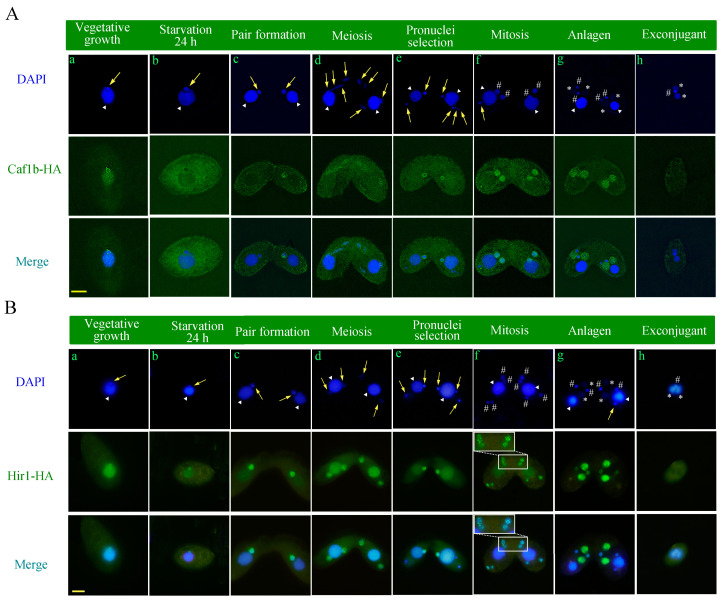
Localization of Caf1b-HA and Hir1-HA during growth, starvation, and conjugation. (**A**) Localization of Caf1b-HA during vegetative growth, starvation, and conjugation. Arrows mark MICs; triangles indicate MACs; # marks zygotic nuclei; * represents new MACs. (**a**) Vegetative growth; (**b**) starvation for 24 h; (**c**) pair formation; (**d**) meiosis; (**e**) pronuclei selection; (**f**) mitosis; (**g**) anlagen; (**h**) exconjugant. Scale bar, 10 μm. (**B**) Localization of Hir1-HA during vegetative growth, starvation, and conjugation. Arrows mark MICs; triangles indicate MACs; # denotes zygotic nuclei; * represents new MACs. (**a**) Vegetative growth; (**b**) starvation for 24 h; (**c**) pair formation; (**d**) meiosis; (**e**) pronuclei selection; (**f**) mitosis. The area inside the white box is magnified 1.5 times at the top. (**g**) Anlagen; (**h**) exconjugant. Scale bar, 10 μm.

Deposition of H3.1 occurs within the replication region, whereas incorporation of H3.3 takes place in the highly transcribed region [45]. To gain a comprehensive understanding of both the RI and RC nucleosome assembly pathways, the localization of Caf1b and Hir1 was investigated in *T. thermophila*. HA-tagged Hir1 and Caf1b constructs were generated (Supplementary Figure S2A,C), which were then transformed into *T. thermophila* via biolistic transformation, followed by selection using paromomycin (Supplementary Figure S2B,D). Indirect immunofluorescence showed that Caf1b-HA localized in the MACs and periphery of MICs in the MIC G2 phase during vegetative growth (Figure 2A(a)). It localized into the MIC and MAC in the MIC S phase and MAC amitosis period (Supplementary Figure S3A(b,c)). After starvation, the signal of Caf1b in the MAC and MIC gradually faded away. Ultimately, Caf1b-HA was localized in the cytoplasm after 24 h of starvation. (Figure 2A(b) and Supplementary Figure S3A(f)). During sexual reproduction, Caf1b-HA localized specifically to meiotic MIC but not in the parental MACs (Figure 2A(c,d)). When the selected pronuclei replicated the DNA and performed prezygotic mitosis, the Caf1b deposition in the MIC was increased (Figure 2A(e)). Caf1b signaling was also found in mitotic MIC (Figure 2A(f)). In the anlagen stage, there was a strong Caf1b signal in the new replicating MAC (Figure 2A(g)). The Caf1b disappeared at the transcribing parental MACs throughout the sexual development stage (Figure 2A(h)).

Hir1-HA localized in the MACs and MICs during vegetative growth (Figure 2B(a), Supplementary Figure S3B). During starvation, Hir1-HA localized in the MAC and the periphery of the MIC (Figure 2B(b), Supplementary Figure S3B). The signaling of Hir1 occurs on both the parental MAC and the new MAC, as well as in both meiotic and mitotic micronuclei (Figure 2B(c–g)). Specifically, Hir1-HA formed a ring structure around pronuclei and new MICs (Figure 2B(e,g)). Surprisingly, Hir1-HA exhibited a punctate signal within expanded postmitotic MIC (Figure 2B(f)). The signal of Hir1 disappeared in the degradable parental MAC at the late sexual development stage (Figure 2B(g)).

### 3.3. Colocalization of Caf1b and Hir1 with Pcna1

**Figure 3:**
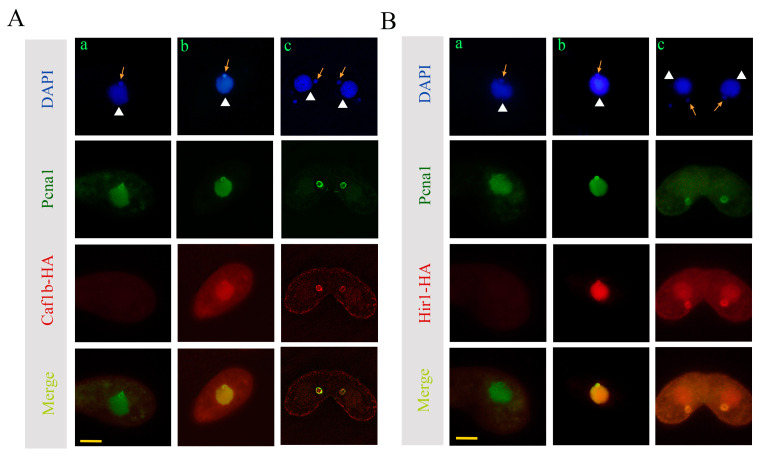
Co-localization of Caf1b-HA and Hir1-HA with Pcna1. (**A**) Colocalization of Caf1b-HA with Pcna1 during vegetative growth and conjugation. (**a**) WT; (**b**,**c**) Caf1b-HA; (**b**) vegetative growth; (**c**) pronuclei selection. (**B**) Co-localization of Hir1-HA with Pcna1. (**a**) WT; (**b**,**c**) Hir1-HA; (**b**) vegetative growth; (**c**) pronuclei selection. Arrows indicate MICs, and triangles mark MACs. Scale bar, 10 μm.

PCNA plays crucial roles in both DNA replication and repair [46,47]. p60 colocalizes with PCNA in HeLa cells [48]. The localization of Hir1 and Caf1b on the MIC was similar but was different in MAC. To further investigate whether Caf1b and Hir1 are involved in the replication progress in *T. thermophila*, colocalization between Pcna1 and Caf1b, as well as between Pcna1 and Hir1, was investigated. Caf1b-HA and Pcna1 colocalized in the MACs and MICs during vegetative growth; furthermore, they co-localized in the MICs during the conjugation stage (Figure 3A(b,c)). Hir1-HA and Pcna1 also colocalized in the MAC and MIC during the vegetative growth stage. They also co-localized in the selected MICs but not MAC during conjugation (Figure 3B(b,c)).

### 3.4. The Absence of HIR1 Resulted in the Loss of Chromatin in MIC

**Figure 4:**
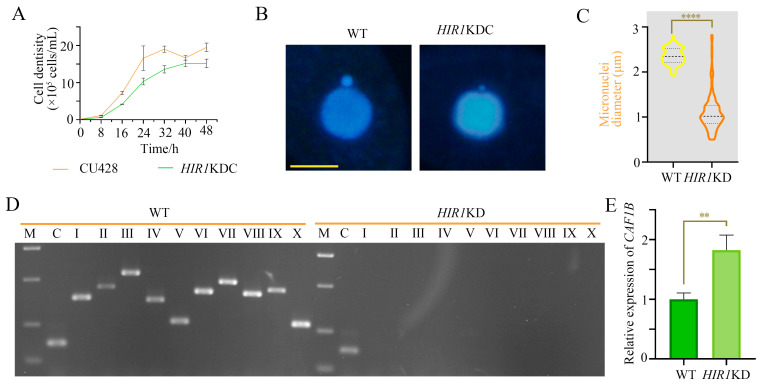
*HIR1* knockdown affected chromatin integrity of MIC. (**A**) Proliferation of *HIR1*KD mutants. Initial cell concentration was 0.125 × 10^5^ cells/mL and cellular proliferation was analyzed every 8 h. (**B**) The nuclear morphology of *HIR1*KD. Scale bar, 10 μm. (**C**) The diameter of MICs in *HIR1*KD. A total of 100 cells were randomly photographed and micronuclear diameters were measured using ImageJ 1.46r. (**D**) Integrity assay of MICs of *HIR1*KD. Gel electrophoresis was performed to detect specific fragments of MICs from WT and *HIR1*KD, which were amplified using 10 pairs of primers. *JMJ1* was utilized as an internal control. M, marker; C, control; I–X, 10 pairs of MIC-specific primers. (**E**) Expression analysis of *CAF1B* was performed in *HIR1*KD mutants and using qRT-PCR. *t*-test was applied for significance analysis, and the expression level of *CAF1B* was significantly upregulated in *HIR1*KD mutant. (** *p* < 0.01; **** *p* < 0.0001).

To further investigate the function of Hir1, the *HIR1* knockout plasmid was constructed and transformed into *T. thermophila* (Supplementary Figure S4A). The *HIR1* knockdown mutants *HIR1*KDB (*HIR1*KD B2086) and *HIR1*KDC (*HIR1*KD CU428) were created, and the expression levels of the mutants down-regulated by 98.82% and 99.70%, respectively (Supplementary Figure S4B,C). *HIR1*KD mutant inhibited cellular proliferation compared to that in the WT (Figure 4A). Observation of nuclear morphology revealed smaller MIC in *HIR1*KD (Figure 4B,C). To further validate our findings, we extracted the genomes from both the mutants and wild-type cells and subsequently amplified micronuclear-specific fragments using 10 pairs of MIC-specific primers. The loss of MIC-specific fragments was observed in *HIR1*KD (Figure 4D). Furthermore, expression of *CAF1B* was up-regulated 1.8-fold in the *HIR1*KD mutants (Figure 4E). These results suggest that Hir1 is involved in the chromatin integrity of MICs.

### 3.5. CAF1B Knockdown Affected Proliferation and Sexual Development of T. thermophila

**Figure 5:**
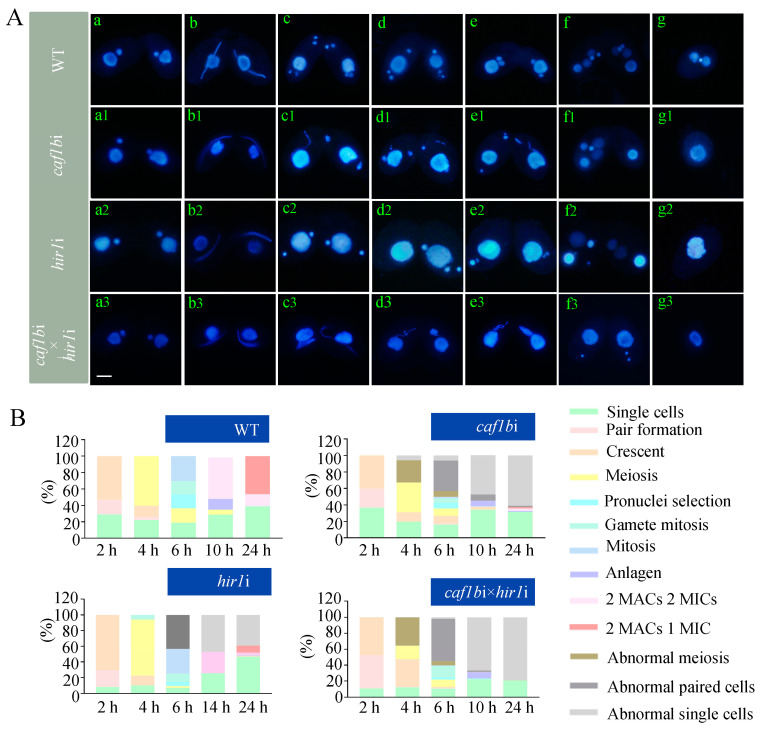
Co-interference of *CAF1B* and *HIR1* affected the meiosis and gametogenesis of *T. thermophila*. (**A**) Nuclear development of wild-type, *caf1b*i, *hir1*i, and *caf1b*i×*hir1*i during conjugation. (**a**–**g**) WT; (**a1**–**g1**) *caf1b*i; (**a2**–**g2**) *hir1*i; (**a3**–**g3**) *caf1b*i×*hir1*i; (**a**,**a1**–**a3**) pair formation; (**b**,**b1**–**b3**) crescent; (**c**,**c2**) meiosis; (**c1**,**c3**,**d1**,**d3**,**e1**,**e3**) abnormal meiosis; (**d**) pronuclei selection; (**e**) mitosis; (**e2**,**f1**–**f3**) abnormal paired cells; (**f**) anlagen; (**g**) exconjugant cells with 2 MACs and 1 MIC; (**g1**–**g3**) abnormal single cells. Cells were stained with DAPI. Cells in the starvation phase were induced with 0.1 μg/mL Cd^2+^ for 48 h, and when cells of different genders were mixed for 2 h, the Cd^2+^ concentration was up-regulated to 0.25 μg/mL. Scale bar, 10 μm. (**B**) Double interference of *CAF1B* and *HIR1* affected the meiosis and gametogenesis of *T. thermophila*. Statistics on the number of cells at each stage during conjugation in wild-type, *caf1b*i, *hir1*i, and *caf1b*i×*hir1*i (*n* = 300).

To investigate the biological function of Caf1b in *T. thermophila*, the *CAF1B* knockout plasmid was constructed and transformed into different mating types *Tetrahymena* (Supplementary Figure S5A). *CAF1B* knockdown mutants *CAF1B*KDB (*CAF1B*KD B2086) and *CAF1B*KDC (*CAF1B*KD CU428) were created (Supplementary Figure S5B). The expression levels of *CAF1B*KDB and *CAF1B*KDC decreased by 98.86% and 93.48%, respectively (Supplementary Figure S5C). To investigate the impact of *CAF1B* deletion, we assessed expression levels of CAF-1’s large subunit (*CAF1A*), small subunit (*REBL1*), *ASF1*, and *HIR1*. The deletion of *CAF1B* led to a down-regulation of expression levels of *CAF1A* (14.83%), *REBL1* (35.36%), and *ASF1* (53.23%); in contrast, *HIR1* showed a 2.28-fold up-regulation (Supplementary Figure S5D–G). Since we want to know the stage-specific function of *CAF1B*, *CAF1B* conditional interference plasmids p*CAF1B*hpNeo5 were constructed and transformed into *T. thermophila* (Supplementary Figure S6A). The expression levels of *CAF1B* in *caf1b*iB and *caf1b*iC under 0.5 μg/mL Cd^2+^ induction for 96 h were reduced by 86.03% and 98.26%, respectively (Supplementary Figure S6B). The proliferation rate of *caf1b*i mutants after being induced using Cd^2+^ decreased than that of WT (Supplementary Figure S6C). To investigate the impact of *CAF1B* on sexual reproduction, we subjected starving cells to 0.1 μg/mL Cd^2+^ treatment for 24 h, and subsequently paired cells of different mating types. After 2 h of pairing, *caf1b*i exhibited a similar phenotype to the wild type, as all cells were capable of reaching their maximum stretching capacity during the crescent period while maintaining intact MIC and MAC (Figure 5A(a,b,a1,b1)). In paired 4 h samples, we observed successful completion of meiosis in the wild type (Figure 5A(c)). Conversely, in the mutants, gradual DNA loss occurred in 27.06% cells during crescent retraction and resulted in a reduction in MIC (Figure 5A(c1–e1),B). After 24 h of mixing, sexual reproduction was completed by 46.42% in the wild-type cells (Figure 5A(g),B), whereas only 1.49% of *caf1b*i cells were capable of forming two MACs and one MIC, and 61.01% of the cells developed abnormal single cells (Figure 5A(g1),B).

### 3.6. Caf1b and Hir1 Are Functionally Complementary and Required for Sexual Reproduction

Caf1b and Hir1 played important roles in the growth and development of *T. thermophila*. To further elucidate the relationship between Caf1b and Hir1, *HIR1* conditional interference mutants were created (Supplementary Figure S6D). Expression levels of *HIR1* in *hir1*iB and *hir1*iC were reduced by 83.25% and 68.89%, respectively (Supplementary Figure S6E). During conjugation, the *hir1*i mutant strain exhibited aberrant pronuclei selection and subsequent mitosis during sexual reproduction (Figure 5A(d2–f2)) and 9.04% cells formed exconjugants with two MACs and one MIC (Figure 5A(g2),B). Subsequently, *caf1b*i and *hir1*i mutants were mixed and initiated sexual development. At 4 h of mixing, 35.65% cells in *caf1b*i×*hir1*i were abnormally meiotic, which is more than *caf1b*i (Figure 5A(c3–e3),B), and none of the cells completed sexual reproduction at 24 h of sexual reproduction (Figure 5A(g3),B). The combined interference of *CAF1B* and *HIR1* led to a more serious phenotype compared to individual interference, which suggests the functional complement of Caf1b and Hir1.

### 3.7. Caf1b and Hir1 Interacted with Different Interactors

**Figure 6:**
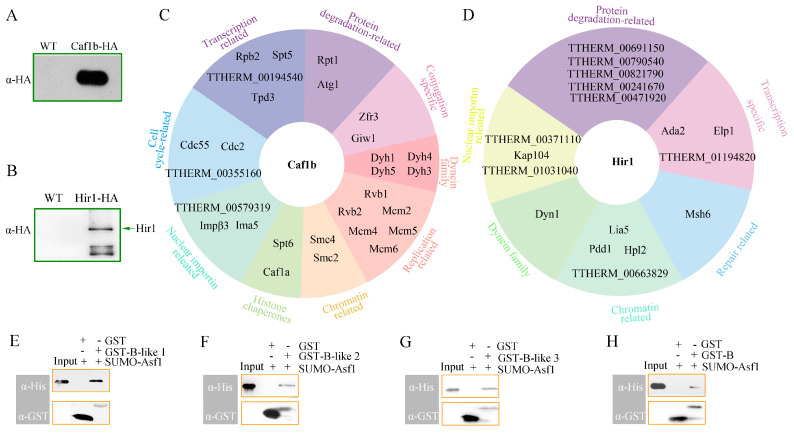
Caf1b and Hir1 interacted with different proteins. (**A**) Western blotting analysis of Caf1b-HA from the eluate. (**B**) Western blotting analysis of Hir1-HA from the eluate. (**C**) Caf1b-HA interaction network. (**D**) Hir1-HA interaction network. (**E**) The interaction between B-like 1 of Caf1b and Asf1. Western blotting was performed using anti-His and anti-GST. (**F**) The interaction between B-like 2 of Caf1b and Asf1. Western blotting was performed using anti-His and anti-GST. (**G**) The interaction between B-like 3 of Caf1b and Asf1. Western blotting was performed using His and GST antibodies. (**H**) The interaction between B domain of Hir1 and Asf1. Western blotting was performed using anti-His and anti-GST.

Although Caf1b and Hir1 interacting proteins were detected previously, specific interactors for Hir1 and Caf1b under different development stages were less clear [49]. To further investigate the function of Caf1b and Hir1, interactors of Caf1b and Hir1 were identified. Given that *CAF1B* and *HIR1* exhibit peak expression during 4–6 h post-pairing, we performed Co-IP analysis using proteins extracted from Caf1b-HA, Hir1-HA, and wild-type cells at 5 h post-pairing. The eluted target proteins following Co-IP were subjected to Western blot analysis, confirming the presence of bait proteins (Figure 6A,B). Subsequent AP-MS assay revealed distinct protein interactions with Caf1b and Hir1. A total of 76 proteins interacting with Caf1b were identified, including histone chaperone Spt6 and Caf1a; proteins specific to chromatin structure Smc2/Smc4; Holliday junction ATP-dependent DNA helicase Rvb1 and Rvb2; DNA replication licensing factor Mcm2, Mcm4, Mcm5, and Mcm6; dynein family proteins; conjugation-specific proteins Giw1 (Gentleman-in-waiting 1) and Zfr3 (Zinc finger-related 3); and proteins related to degradation, transcription, cell cycle, and nuclear import (Figure 6C).

We identified 56 proteins that interacted with Hir1, mainly including pdd1 (programmed DNA degradation 1); heterochromatin protein 1 like 2 (Hpl2); transposase Lia5 (localized in macronuclear anlagen 5); class I histone deacetylase family protein (TTHERM_00663829); DNA mismatch repair protein (Msh6); transcription-related protein Ada2 (transcriptional adaptor 2), Elp1 (elongation protein 1), and TTHERM_011942820; proteins related to degradation and nuclear importin, and dynein family protein (Figure 6D).

### 3.8. Caf1b and Hir1 Interacted with Asf1 In Vitro

Homologous proteins of Caf1b and Hir1 were found to interact with Asf1 in both mammals and yeast [19,27]. However, the mass spectrometry results of Caf1b-HA and Hir1-HA did not reveal the presence of Asf1. To investigate the interaction between Asf1 and Caf1b/Hir1 in *T. thermophila*, we constructed fusion proteins by ligating the three predicted B-domain-like motifs of Caf1b and the B domain of Hir1 into the pGEX-6P-1 expression vector, while Asf1 was ligated to a SUMO vector for expression in *E. coli*. The interaction analysis between Caf1b/Hir1 and Asf1 was performed using a pull-down assay. Western blot and SDS-PAGE analyses revealed that each of the three B-domain-like motifs of Caf1b interacts with Asf1 (Figure 6E–G, Supplementary Figure S7A–C). These findings demonstrate that the binding between Caf1b and Asf1 is mediated by multiple sites. Similarly, Hir1 could interact directly with Asf1 in vitro (Figure 6H, Supplementary Figure S7D). The results indicate that both Caf1b and Hir1 interact directly with Asf1.

## 4. Discussion

The precise wrapping of histones by DNA is crucial for maintaining genome stability and ensuring the accurate transmission of genetic information. CAF-1 and Hir1 play pivotal roles as conserved histone chaperones in eukaryotes, facilitating the deposition of histone variants H3.1 and H3.3 onto DNA, respectively. This process not only enables the efficient compaction of genomic DNA within the nuclear space but also actively participates in gene expression regulation. The functional conservation and diversity of CAF-1 and Hir1 have been observed across different eukaryotes [50,51,52]. In this study, we demonstrated that Caf1b and Hir1 exhibit similar MAC and MIC localization patterns during vegetative growth. Hir1 localized in the parental MACs; however, the Caf1b signal disappeared in the parental MACs during sexual reproduction. Following *CAF1B* knockdown, *HIR1* expression up-regulated. Similarly, after *HIR1* knockdown, there was an up-regulated expression of *CAF1B*, which suggests a complementary relationship between Caf1b and Hir1. In addition, both Caf1b and Hir1 play important roles in cell proliferation and sexual reproduction.

### 4.1. Similar and Different Dynamic Distribution Patterns of Caf1b and Hir1 in T. thermophila

*CAF1B* and *HIR1* had low expression levels during growth and starvation but showed a significant increase in expression at 4–6 h of conjugation. In budding yeast, CAF-1 is localized to centromeres throughout the cell cycle [23]. In mammalian cells, p60 binds to the nucleus during interphase, dissociates from chromatin during mitosis, and concentrates in the nucleus at sites of DNA replication during the S-phase [53]. During vegetative growth, Caf1b-HA localized to both the MACs and MICs during growth; however, the nuclear signal disappeared and a cytoplasmic signal increased at starving 24 h in *T. thermophila* (Figure 2A(a,b)). Caf1b-HA only localized in the MICs during meiosis and mitosis, the periphery of MICs during post-meiotic nuclear selection, and the new MACs during the anlagen stage (Figure 2A(c–g)). Additionally, Caf1b-HA colocalized with Pcna1 during vegetative growth and conjugation (Figure 3A(b,c)). During vegetative growth, MAC undergoes amitosis, while MIC undergoes mitosis during asexual reproduction, indicating the involvement of Caf1b in DNA replication in both MACs and MICs. Prior to micronuclear stretching, DNA double-strand breaks occur [54]. Caf1b was localized to meiotic MIC, which suggests it might be involved in post-DSB repair. One selected meiotic product proceeds through mitosis while the remaining nuclei degenerate [55]. The selected gamete nuclei undergo DNA replication and chromatin remodeling [56]. The strong signal of Caf1b-HA at the selected MICs suggests it may involve in DNA replication and gamete remodeling. The zygotic nuclei undergo two mitosis and two of the postzygotic mitotic products develop into new MACs [37,57]. Caf1b-HA localized in the mitotic zygotic nucleus and the anlagen but disappeared in the parental MAC. The different localization implied that Caf1b might be involved in the replication and remodeling of MICs and anlagen. Furthermore, Caf1b also colocalized with Pcna1 in a different development stage, which suggests that CAF-1 participates in the chromatin replication in *T. thermophila*. Heterochromatin was predominantly localized in the perinuclear region [58]. Hir1 and Caf1b also localized to the nuclear periphery of the MIC during the G2 phase. Hir1 also localized to the periphery of pronuclei and new MICs. They might be stored in the nucleoplasm. Another possibility is that Hir1 and Caf1b associate with heterochromatin regions and participate in chromatin remodeling processes. The exact mechanisms and functions of Hir1 and Caf1b in these contexts require further investigation.

In human cells, HIRA localizes in the nucleus [59]. In Mesenchymal stem cells, HIRA colocalized with H3.3 at promyelocytic leukemia (PML) bodies [60]. In *T. thermophila,* Hir1 localized to the MACs and MICs during asexual reproduction and starvation (Figure 2B(a,b), Supplementary Figure S3B). In sexual reproduction, Hir1 was found in transcriptionally active parental MACs at an early conjugation stage (Figure 2B(c–f)), providing direct evidence for its association with transcription progress. Surprisingly, Hir1 was also observed to localize to meiotic and mitotic MICs during sexual reproduction (Figure 2B(c–f)), and co-localization with Pcna1 on the selected pronuclei (Figure 3B(c)). The results imply that Hir1 is involved in replication-related processes or chromatin remodeling. Interestingly, punctate signals were observed on the enlarged postmitotic MICs at early anlagen stage. The localization of Hir1-HA is similar to that of Cna1 on the expanded MICs [61,62]. In mammalian cells, H3.3 serves as a placeholder for CENP-A during the assembly of centromeric nucleosomes [63]. In *T. thermophila*, the formation of new MACs involves IES elimination after the replication of MICs from 4C to 8C, with no presence of Cna1 in MACs [62]. Therefore, we speculate that Hir1 might be involved in Cna1 replacement and centromeric deletion.

It is reported that the deposition of H3.1 occurs within the replication region, whereas the incorporation of H3.3 takes place in the highly transcribed region [45]. GFP-H3.1 is localized to the MAC and MIC in *T. thermophila*, whereas GFP-H3.3 is localized only to the persistently transcriptionally active MAC. The parental MAC is only transcribed and not replicated during sexual reproduction. Combining the localization of GFP-H3.3 and GFP-H3.1 with that of Caf1b-HA and Hir1-HA, we hypothesized that CAF-1 could be involved in RC-nucleosome-assembly-mediated deposition of H3.1, whereas Hir1 mediates the RI-nucleosome-assembly-mediated deposition of H3.3.

### 4.2. Caf1b and Hir1 Are Required for Proliferation and Sexual Reproduction of T. thermophila

In human cells, silencing of Caf1b/p60 leads to cell death and loss of chromatin assembly [64]. Deletion of caf-1b in zebrafish led to S-phase arrest and impaired differentiation [65]. In the chicken DT40 B-cell line, depletion of Caf1b led to delayed S-phase progression with retarded DNA synthesis and defects in a rapid nucleosome formation of newly replicated DNA, and cell death [20]. However, the deletion Cac2/Caf1b in *Saccharomyces cerevisiae* exhibits a minimal effect on growth. Knockdown of *CAF1B* in *T. thermophila* inhibited cellular proliferation during vegetative growth (Supplementary Figure S6C) and disturbed meiotic progress (Figure 5A(c1–e1)). There were Caf1b signals on the meiotic MIC. Moreover, proteins interacted with Caf1b including Dyh1, Dyh3, Dyh4, and Dyh5 interactors associated with microtubule movement, chromosome movement, and distribution. Chromosome loss during retraction after stretching of the MIC to its maximum length upon deletion of *CAF1B* may be due to abnormal microtubule movement during MIC retraction, which leads to chromosome breakage and degradation or the abnormal repair of DSBs.

HIRA is indispensable for H3.3 deposition in mouse zygotes, and its deletion hinders the de novo assembly of nucleosomes [66]. It was surprising to us that the absence of *HIR1*, a key element in RI nucleosome assembly, resulted in compromised MIC integrity. In *T. thermophila*, H3.3 weakly signals on the transcriptionally silent MIC via the inefficient RC pathway [38]. Impairment of CAF-1-mediated deposition of H3.1 in HeLa cells promotes the deposition of H3.3 on DNA with the assistance of HIRA [5]. In *T. thermophila*, we observed an up-regulation of *HIR1* in the *CAF1B*KD mutants, and up-regulation of *CAF1B* was also observed in *HIR1*KD mutants (Supplementary Figure S5G, Figure 4E). Therefore, there is a complementary function between Caf1b and Hir1. Removal of Hira leads to the rapid decline of hematopoietic stem cells [67]. Simultaneous deletion of CHAF1B and HIRA renders cells more susceptible to DNA damage in leukemic cells [25]. In yeast, the cac1 and hir1 double-mutant cells exhibit a broad distribution of centromere histone Cse4 outside the centromere [68]. In *Tetrahymena*, co-interference of *HIR1* and *CAF1B* resulted in a more serious aberrant meiotic phenotype than individual mutants (Figure 5A,B).

### 4.3. Caf1b and Hir1 Interacted with Different Proteins

The interaction between Asf1 and Caf1b has been observed in *Drosophila*, *Arabidopsis*, and yeast [69]. Caf1b contains B-domain-like motifs that bind to Asf1 [70]. Caf1b contains two tandem B-domain-like motifs at its C-terminal in mammalian cells, whereas only one B domain is present in *D. melanogaster* and *S. cerevisiae* [19]. However, Caf1b contained three B-domain-like motifs in *Tetrahymena*. The three B-domain-like motifs of Caf1b directly interacted with Asf1 in vitro. Furthermore, distinct B-domain-like motifs also indicate subtle structural variations between Caf1b and its homologous counterparts. In *T. thermophila*, the interaction between Caf1b and Caf1a was identified but not RebL1, indicating a more intensive interaction with Caf1a compared to RebL1, which may be attributed to the fact that the intermediate subunit interacts with the small subunit through the large subunit. In U2OS and HeLa cells, the chromodomain Y-like protein (CDYL) mediates the interaction between CAF-1 and MCM complexes, facilitating the transfer and deposition of histones during DNA replication [71]. In HeLa S3 cells, the enrichment of CAF-1 complexes comprising all three subunits (p150, p60, and p48) was observed in eH3.1 and eH3.2 immunoprecipitations (IPs), while MCM2, 4, 6, and 7 exhibited interactions with all H3 variants [72]. In *T. thermophila*, we identified direct or indirect interactions between Caf1b and members of the MCM protein family (Figure 6C). Caf1b exhibitsed co-localization with Pcna1 and interacted with a diverse range of proteins involved in replication, cell cycle regulation, and ubiquitination-mediated protein degradation, indicating its role as a replication-associated protein regulated via the cell cycle.

In mammals, UBN1, CABIN1, and HIRA form a stable HIR complex. In yeast, the complex is composed of Hir1, Hir2, Hir3, and Hpc2 [27,32]. However, we did not find a similar subunit of HIR complexes that interact with Hir1 in *Tetrahymena*. We also failed to identify homologs of UBN1 and CABIN1 using a bioinformatics method in *Tetrahymena*. We hypothesized that the Hir1 complex could form a different assembly or have a different composition compared to other organisms. Further experimental investigation is required to gain a better understanding of the specific composition and functions of the Hir1 complex. Normal expression of Hira in the mouse is essential for transcriptional regulation [35]. In *Plasmodium falciparum*, HIR is located in the heterochromatin region [73]. Hir1 interacted with the chromodomain proteins Hpl2 and Pdd1, and formed punctate structure in the early anlagen stage (Figure 2B(f) and Figure 6D), which implies that Hir1 is involved in heterochromatin formation in *T. thermophila*. The interaction of Hir1 and Caf1b with distinct proteins and their formation of different complexes suggests their functional diversity and complexity.

## 5. Conclusions

Caf1b and Hir1 contain conservative WD40 domains and B domains and have similar expression profiles. Both played important roles in the sexual and asexual reproduction of *T. thermophila*. Although Caf1b and Hir1 formed different complexes, they exhibited functional complementarity for nucleosome assembly. These findings in *Tetrahymena* deepen the understanding of RC and RI nucleosome assembly mechanisms.

## Data Availability

All relevant data are within the paper and its additional files. The data used to support the findings of this study are available upon reasonable request.

## Supplementary Materials

The following supporting information can be downloaded at https://www.mdpi.com/article/10.3390/cells12242828/s1. Figure S1: B-domain-like motifs and B domain of Caf1b and Hir1 in different organisms. Figure S2: Identification of Caf1b-HA and Hir1-HA. Figure S3: Localization of Caf1b-HA and Hir1-HA during vegetative growth and starvation. Figure S4: Identification of *HIR1*KD cells. Figure S5: The identification of *CAF1B* knockdown mutants. Figure S6: Construction and identification of *CAF1B* and *HIR1* conditional knockdown mutants. Figure S7: Caf1b and Hir1 interacted with Asf1 in vitro. Table S1: Primer used in the work. Table S2: Mass spectrometry results of Caf1b-HA during conjugation. Table S3: Mass spectrometry results of Hir1-HA during conjugation.
