# Supplementary material for: Independent and Complementary Functions of Caf1b and Hir1 for Chromatin Assembly in Tetrahymena thermophila
Source: Cells. 2023 Dec 13;12(24):2828. doi: 10.3390/cells12242828 (PMC10741905; doi:10.3390/cells12242828)
Supplement: Supplementary file 1 [file cells-12-02828-s001.zip › cells-2751267 supplementary material.pdf]

Independent and Complementary Functions of Caf1b and Hir1 for Chromatin Assembly in *Tetrahymena thermophila*

Huijuan Hao [1], Chenhui Ren [2], Yinjie Lian [3], Min Zhao [4], Tao Bo [5], Jing Xu [1,2], and Wei Wang [1,3,*]

Supplementary information:

Supplementary Figures S1-S7

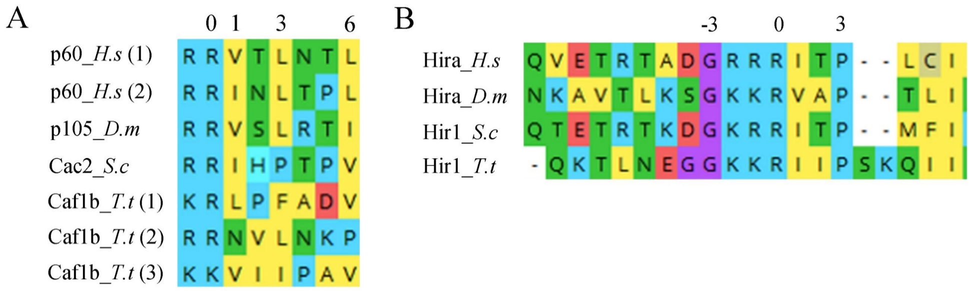

Figure S1. B-domain-like motifs and B domain of Caf1b and Hir1 in different organisms. (**A**) The alignment of the sequence of the B-domain-like motif of the Caf1b homologous. The conserved arginine residue (R) is designated as position 0. Subsequently, the amino acids at positions 1, 3, and 6 are also highly conserved and all exhibit hydrophobic properties. (**B**) Sequence alignment of the B domain of the Hir1 homolog. The conserved arginine residue is denoted as 0. Both the amino acid at position -3 and the amino acid at position 3 are also highly conserved.

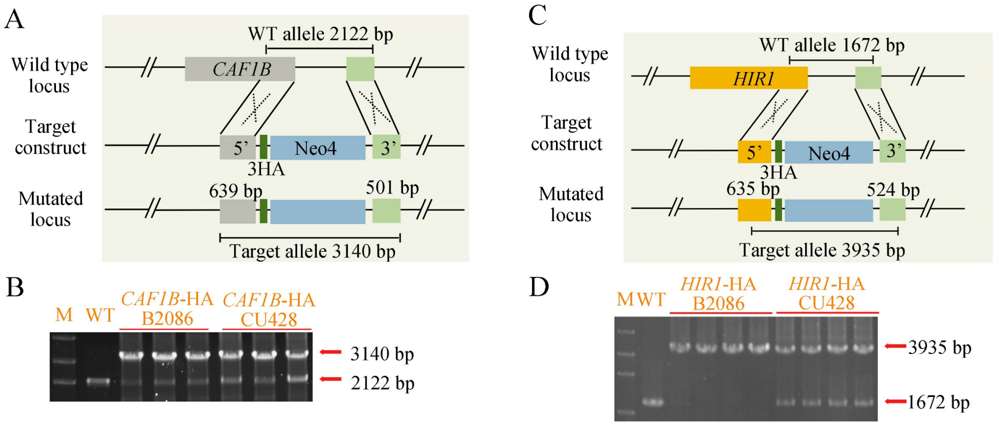

Figure S2. Identification of Caf1b-HA and Hir1-HA. (**A**) Diagram of the construction of *CAF1B*-HA plasmid. The elongated gray rectangle represents the *CAF1B*, while the shorter gray rectangles denote the fragment before its stop codon. The dark green rectangles signify three tandem HA tags, and the blue rectangles indicate the Neo4 cassette. Additionally, the light green rectangles represent the 3' flanking sequence of *CAF1B*. (**B**) Identification of the homologous recombination substitution of *CAF1B*-HA. The genome of the mutant strains and WT was amplified by PCR with primers shoot-*CAF1B*-HA-F/R. Arrows

mark wild-type (2122 bp) and mutant locus (3140 bp). (**C**) Diagram of the construction of *HIR1*-HA plasmid. The elongated orange rectangle represents the *HIR1*, while the shorter orange rectangles denote the fragment before its stop codon. The dark green rectangles signify three tandem HA tags, and the blue rectangles indicate the Neo4 cassette. Additionally, the light green rectangles represent the 3' flanking sequence of the target gene. (**D**) Identification of the homologous recombination substitution of *HIR1*-HA. The genome of the mutant strains and WT was amplified by PCR with primers shoot-*HIR1*-HA-F/R. Arrows mark wild-type (3935 bp) and mutant locus (1672 bp).

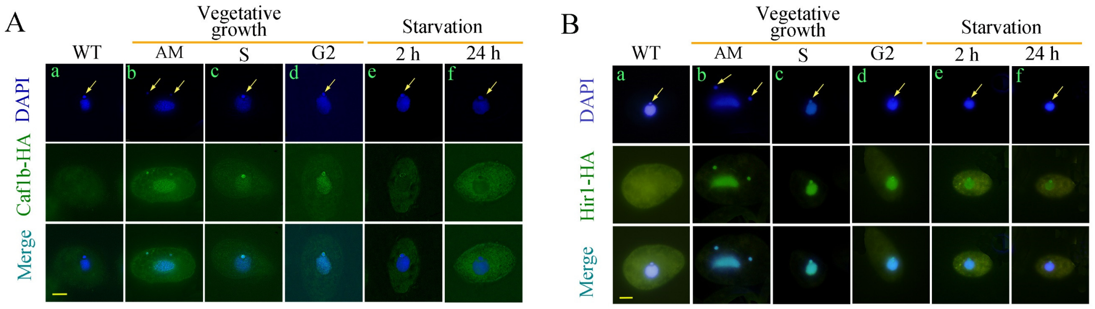

Figure S3. Localization of Caf1b-HA and Hir1-HA during vegetative growth and starvation. (**A**) Localization of Caf1b-HA during vegetative growth and starvation. Arrows indicate MICs. (**a**) WT; (**b–f**) Caf1b-HA; (b), MAC amitosis; (**c**) MIC S phase; (**d**) MIC G2 phase same as Figure 2A (a); (e), starvation for 2 h; (**f**) starvation for 24 h same as Figure 2A (b). Scale bar, 10 µm. (**B**) Localization of Hir1-HA during vegetative growth and starvation. Arrows indicate MICs. (**a**) WT; (**b–f**) Hir1-HA; (b), MAC amitosis; (**c**) MIC S phase; (**d**) MIC G2 phase same as Figure 2B (a); (e), starvation for 2 h; (**f**) starvation for 24 h same as Figure 2B (b). Scale bar, 10 µm.

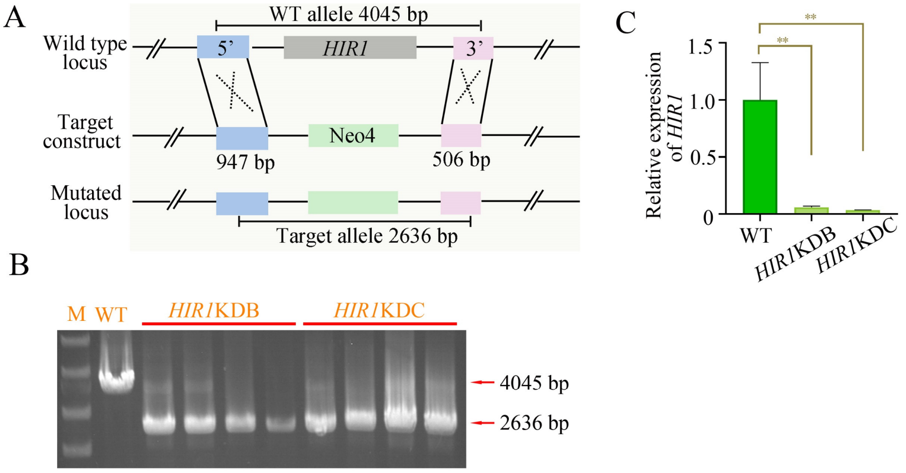

Figure S4. Identification of *HIR1*KD cells. (**A**) Schematic diagram of plasmid construction for knockdown of *HIR1*. The gray rectangle represents *HIR1*, while the green rectangles denote the Neo4 cassette. Additionally, the blue rectangles denote the 5' flanking sequence of *HIR1*, and the pink rectangles represent its 3' flanking sequence. (**B**) Identification of *HIR1*KD mutants. Arrows mark wild-type (4045 bp) and mutant locus (2636 bp). (**C**) Expression of *HIR1* in *HIR1*KD mutants. RNA was extracted from WT and *HIR1*KD cells. Identifying the expression level of *HIR1* was analyzed by qRT-PCR. t-test was applied for significance analysis (** $p < 0.01$).

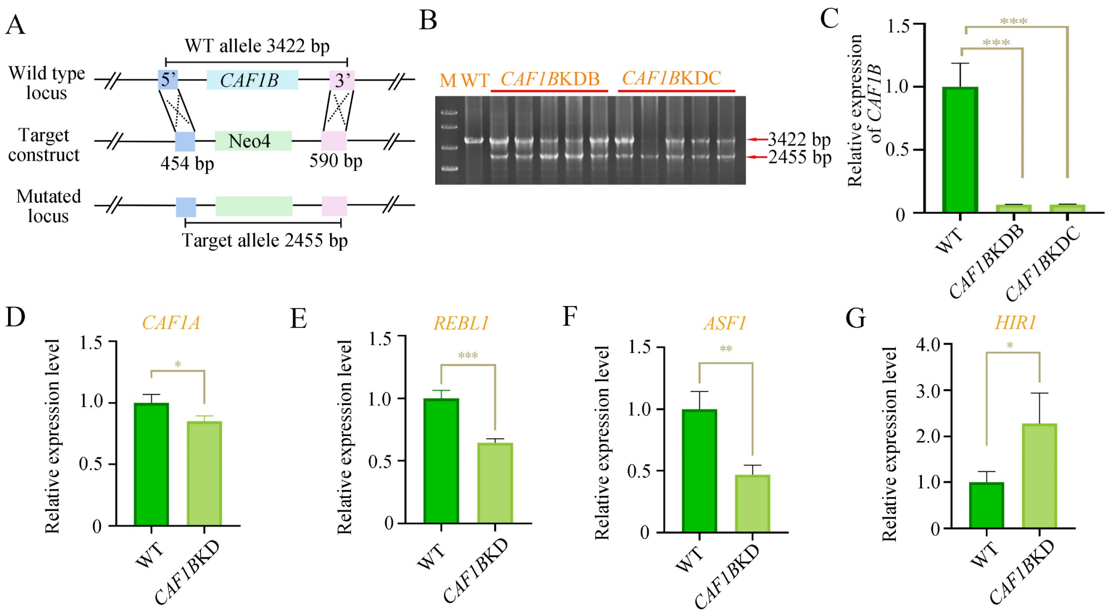

Figure S5. The identification of *CAF1B* knockdown mutants. (**A**) Schematic diagram of plasmid construction for knockdown of *CAF1B*. The light blue rectangle represents *CAF1B*, while the green rectangles denote the Neo4 cassette. Additionally, the dark blue rectangles denote the 5' flanking sequence of *CAF1B*, and the pink rectangles represent its 3' flanking sequence. (**B**) Identification of *CAF1B*KD mutants. Arrows indicate wild-type (3422 bp) and mutant locus (2455 bp). (**C**) Expression level of *CAF1B* in *CAF1B*KD mutants. RNA was extracted from WT and *CAF1B*KD cells. Identifying the expression level of *CAF1B* by qRT-PCR. t-test was applied for significance analysis (*** $p < 0.001$). (**D–G**) Expression analysis of *CAF1A*, *REBL1*, *ASF1*, and *HIR1* was performed in *CAF1B*KD mutants and using qRT-PCR. t-test was applied for significance analysis (* $p < 0.05$, ** $p < 0.01$, *** $p < 0.001$).

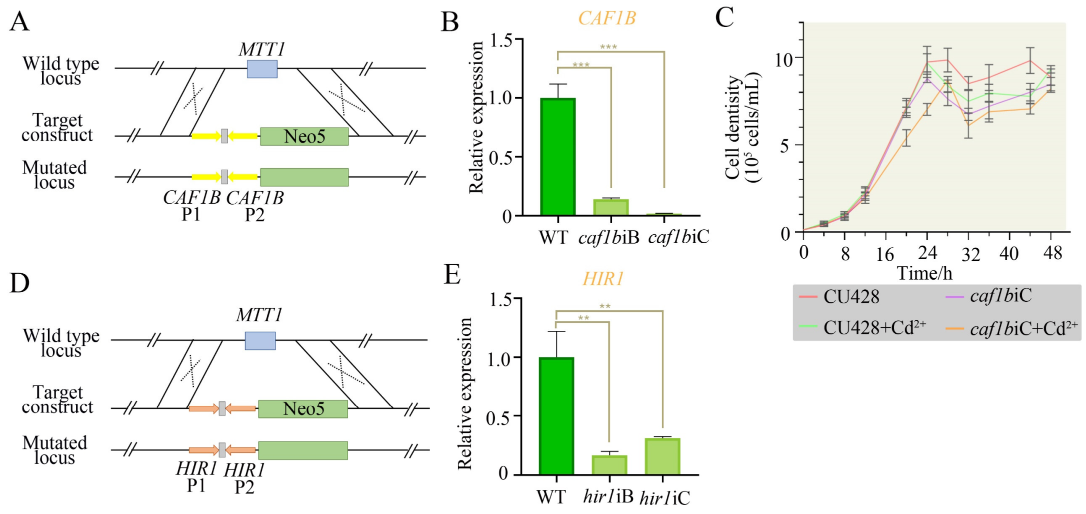

Figure S6. Construction and identification of *CAF1B* and *HIR1* conditional knockdown mutants. (**A**) Schematic diagram of p*CAF1B*hpNeo5. The blue rectangle represents *MTT1*, while the yellow arrows represent the same fragment in the *CAF1B* that is conservatively oriented differently. The linker between these two arrows is illustrated as a gray rectangle, and the Neo5 cassette is denoted by green rectangles. (**B**) Expression level of *CAF1B* in *caf1b*i mutants. The cells induced with 0.5 μg/mL Cd^{2+} for 96 hours during growth were collected. *CAF1B* expression levels were assessed using qRT-PCR with RT-*CAF1B*-F/R

primers. t-test was applied for significance analysis (*** $p < 0.001$). (**C**) The proliferation of *caf1b*i mutant. The initial cell count was 0.125×10^5 cells/mL. *caf1b*i and CU428+Cd^{2+} were induced with 0.5 µg/mL Cd^{2+} for 96 hours. (**D**) Schematic diagram of p*HIR1*hpNeo5. The blue rectangle represents *MTT1*, while the orange arrows represent the same fragment in the *HIR1* that is conservatively oriented differently. The linker between these two arrows is illustrated as a gray rectangle, and the Neo5 cassette is denoted by a green rectangle. (**E**) The expression level of *HIR1* in *hir1*i mutants. RNA was extracted from WT and *hir1*i mutants induced with 0.5 µg/mL Cd^{2+} for 96 h. The expression level of *HIR1* was analyzed by qRT-PCR with primer RT-*HIR1*-F/R. t-test was applied for significance analysis (** $p < 0.01$).

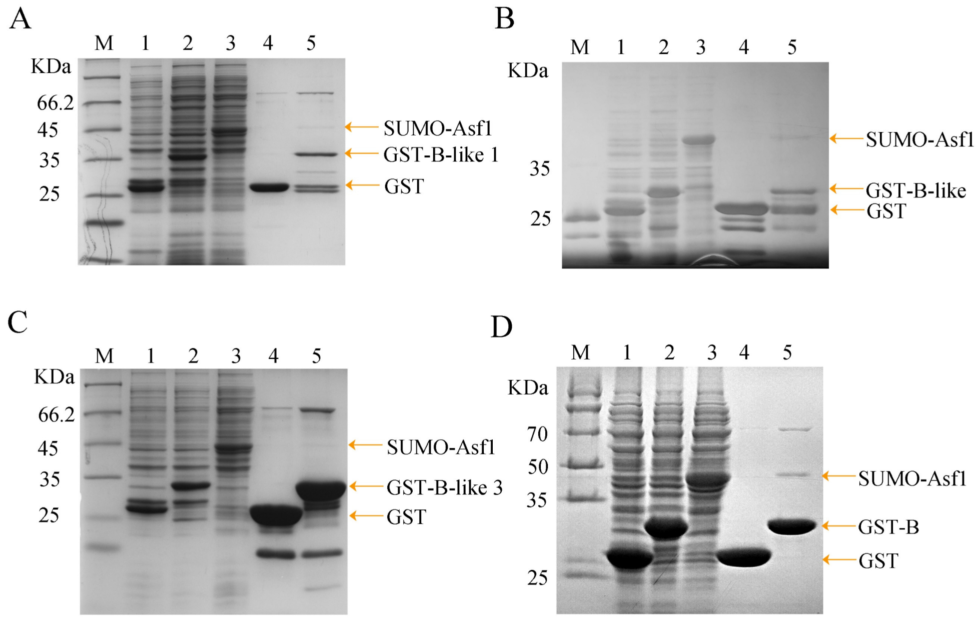

Figure S7. Caf1b and Hir1 interacted with Asf1 in vitro. (**A**) SDS-PAGE analysis of GST-B-like 1 and SUMO-Asf1 interactions. M, Marker; 1, supernumerary of GST; 2, supernumerary of GST-B-like 1; 3, supernumerary of SUMO-Asf1; 4, eluent from GST and SUMO-Asf1; 5, eluent from GST-B-like 1 and SUMO-Asf1. (**B**) SDS-PAGE analysis of GST-B-like 2 and SUMO-Asf1 interactions. M, Marker; 1, supernumerary of GST; 2, supernumerary of GST-B-like 2; 3, supernumerary of SUMO-Asf1; 4, eluent from GST and SUMO-Asf1; 5, eluent from GST-B-like 2 and SUMO-Asf1. (**C**) SDS-PAGE analysis of GST-B-like 3 and SUMO-Asf1 interactions. M, Marker; 1, supernumerary of GST; 2, supernumerary of GST-B-like 3; 3, supernumerary of SUMO-Asf1; 4, eluent from GST and SUMO-Asf1; 5, eluent from GST-B-like 3 and SUMO-Asf1. (**D**) SDS-PAGE analysis of GST-B and SUMO-Asf1 interactions. M, Marker; 1, supernumerary of GST; 2, supernumerary of GST-B; 3, supernumerary of SUMO-Asf1; 4, eluent from GST and SUMO-Asf1; 5, eluent from GST-B and SUMO-Asf1.
