# Supplementary material for: Independent and Complementary Functions of Caf1b and Hir1 for Chromatin Assembly in Tetrahymena thermophila
Source: Cells. 2023 Dec 13;12(24):2828. doi: 10.3390/cells12242828 (PMC10741905; doi:10.3390/cells12242828)
Supplement: Supplementary file 1 [file cells-12-02828-s001.zip › Original Images.pdf]

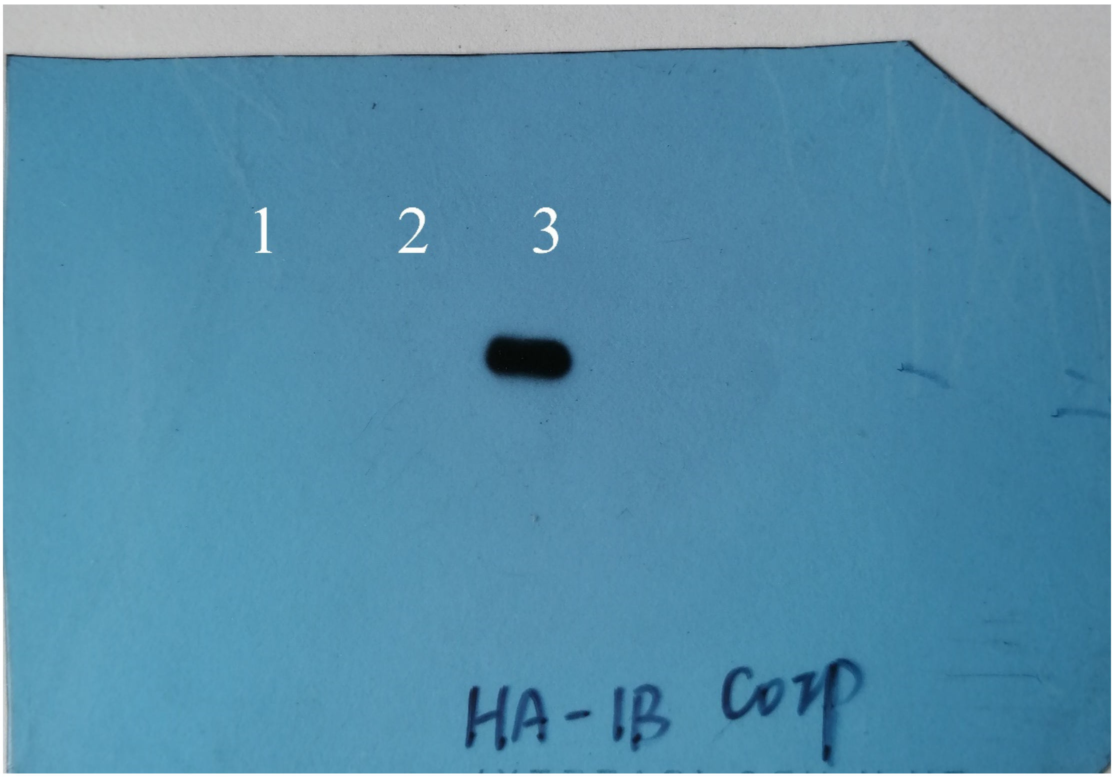


This figure shows the original data for Figure 6A. The order of lane loading from left to right was as follows: lane 1: Marker; lane 2: WT; lane 3: Caf1b-HA.

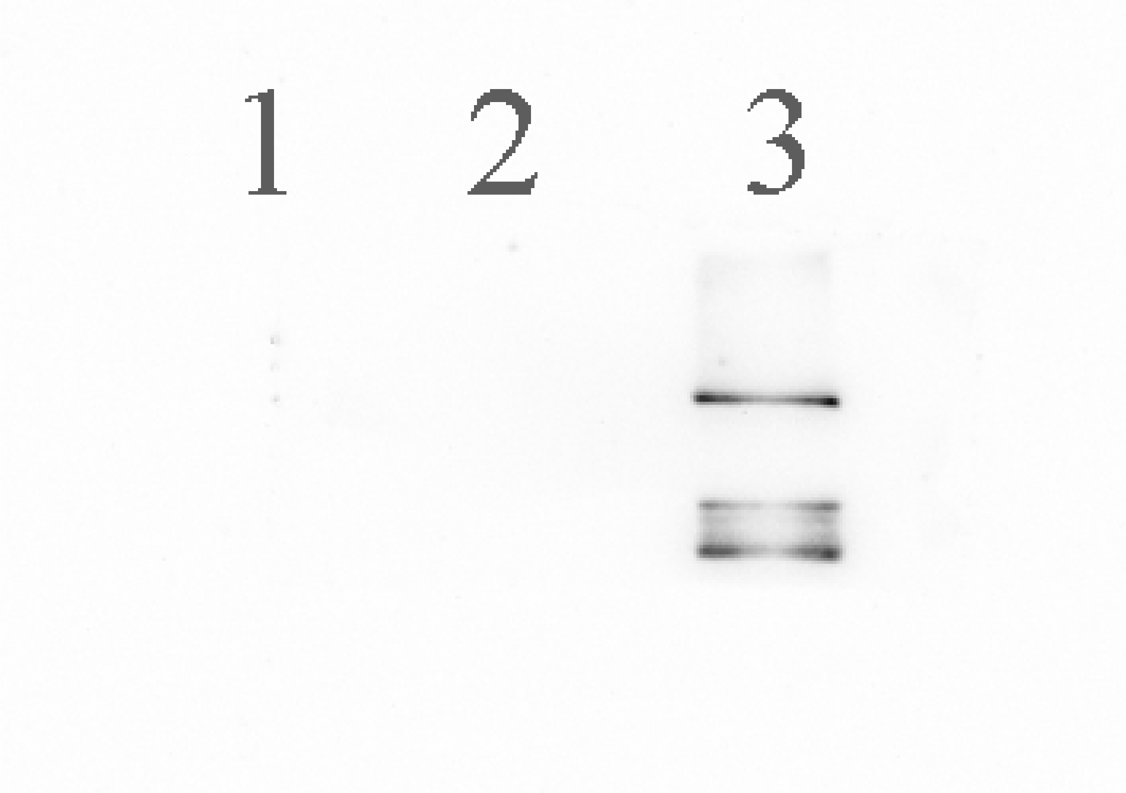


This figure shows the original data for Figure 6B. The order of lane loading from left to right was

as follows: lane 1: Marker; lane 2: WT; lane 3: Hir1-HA.

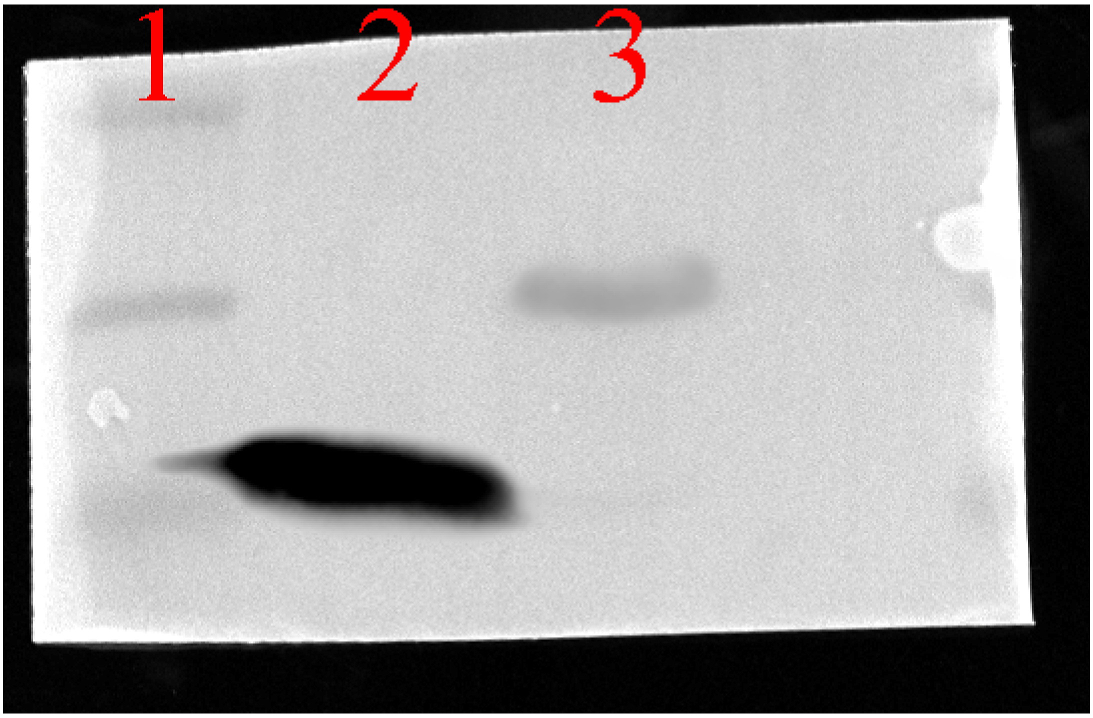


This figure shows the original data for Figure 6E. The order of lane loading from left to right was as follows: lane 1: Marker; lane 2: The supernatants of GST and SUMO-Asf1 were passed through a GST gel column and then eluted with 10 mM GSH, which was detected by GST antibody; lane 3: The supernatants of GST-B-like 1 and SUMO-Asf1 were passed through a GST gel column and then eluted with 10 mM GSH, which was detected by GST antibody.

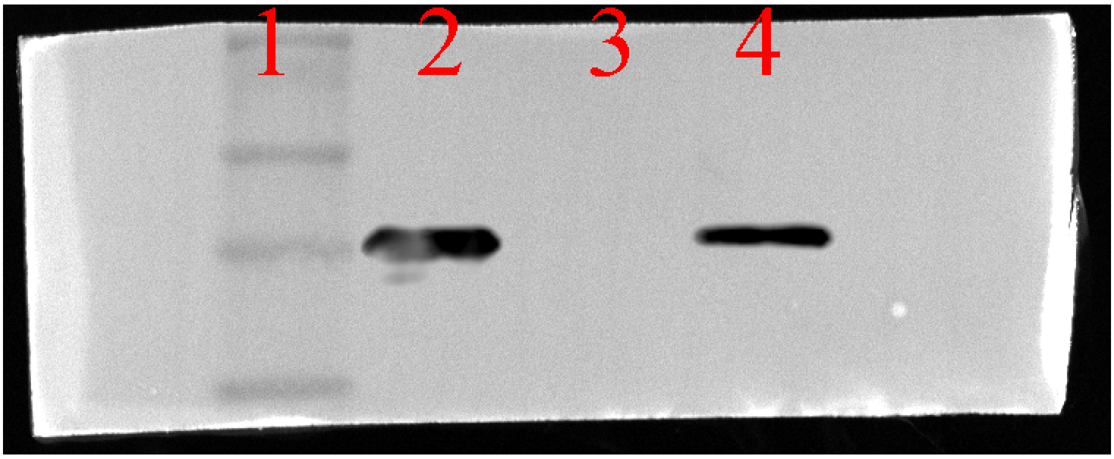


This figure shows the original data for Figure 6E. The order of lane loading from left to right was as follows: lane 1: Marker; lane 2: The supernatants of SUMO-Asf1. lane 3: The supernatants of GST and SUMO-Asf1 were passed through a GST gel column and then eluted with 10 mM GSH, which was detected by His antibody; lane 4: The supernatants of GST-B-like 1 and SUMO-Asf1 were passed through a GST gel column and then eluted with 10 mM GSH, which was detected by His antibody.

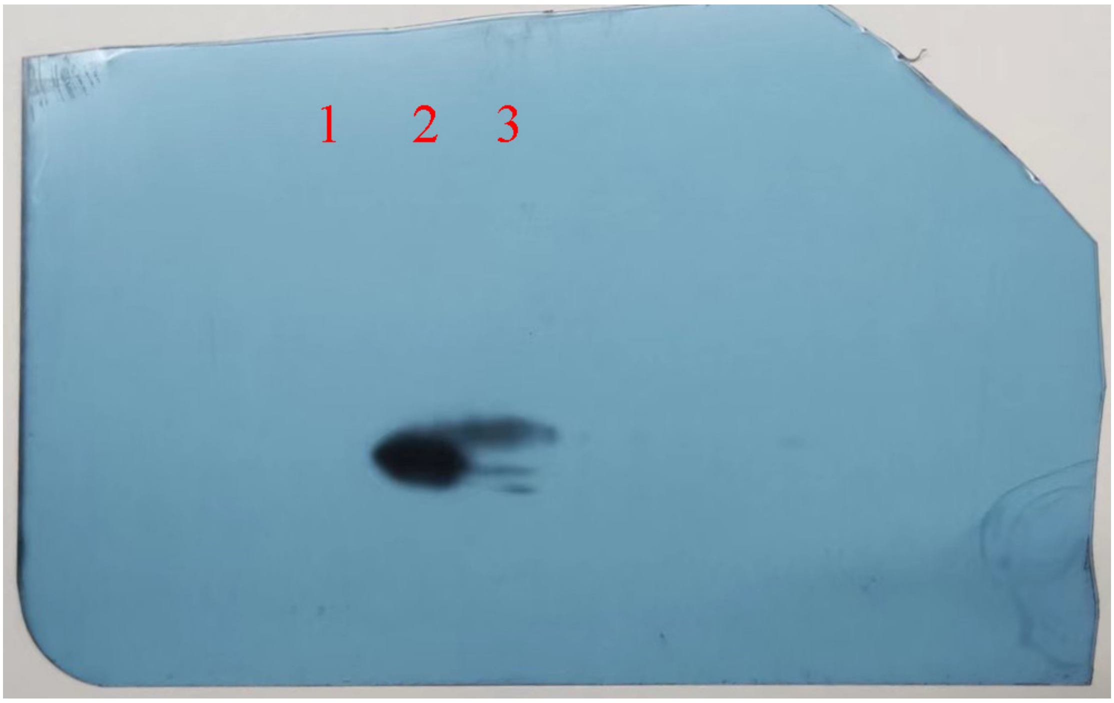


This figure shows the original data for Figure 6F. The order of lane loading from left to right was as follows: lane 1: Marker; lane 2: The supernatants of GST and SUMO-Asf1 were passed through a GST gel column and then eluted with 10 mM GSH, which was detected by GST antibody; lane 3: The supernatants of GST-B-like 2 and SUMO-Asf1 were passed through a GST gel column and then eluted with 10 mM GSH, which was detected by GST antibody.

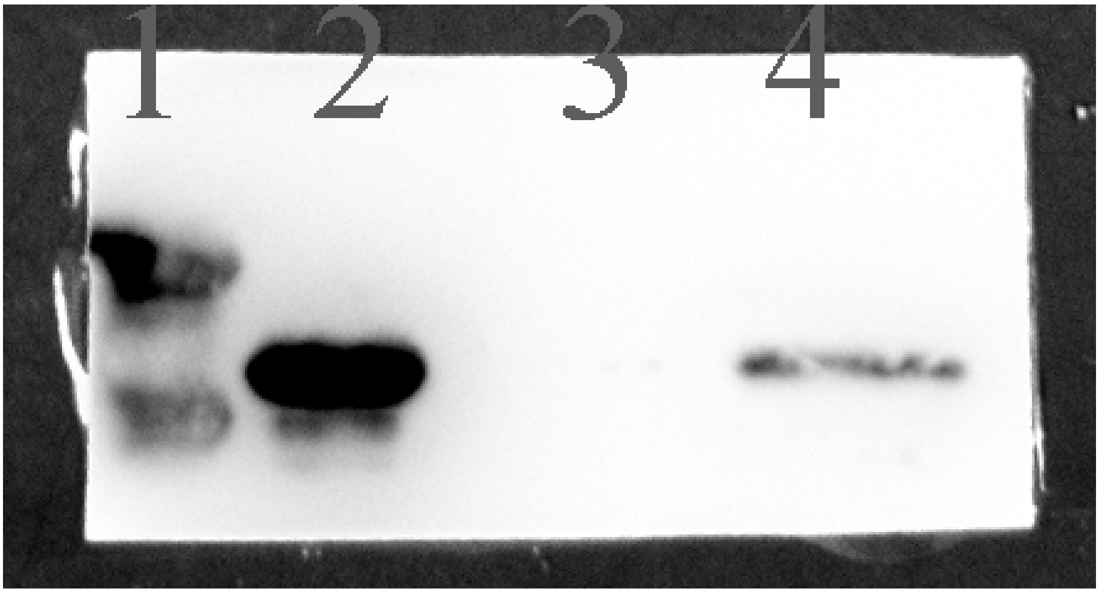


This figure shows the original data for Figure 6F. The order of lane loading from left to right was as follows: lane 1: Marker; lane 2: The supernatants of SUMO-Asf1. lane 3: The supernatants of GST and SUMO-Asf1 were passed through a GST gel column and then eluted with 10 mM GSH, which was detected by His antibody; lane 4: The supernatants of GST-B-like 2 and SUMO-Asf1 were passed through a GST gel column and then eluted with 10 mM GSH, which was detected by His antibody.

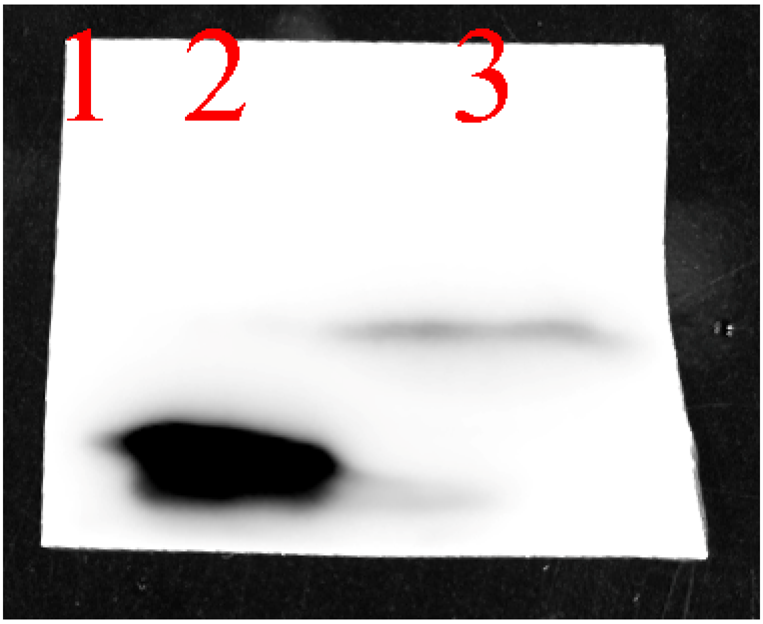


This figure shows the original data for Figure 6G. The order of lane loading from left to right was as follows: lane 1: The supernatants of GST and SUMO-Asf1 were passed through a GST gel column and then eluted with 10 mM GSH, which was detected by GST antibody; lane 3: The supernatants of GST-B-like 3 and SUMO-Asf1 were passed through a GST gel column and then eluted with 10 mM GSH, which was detected by GST antibody.

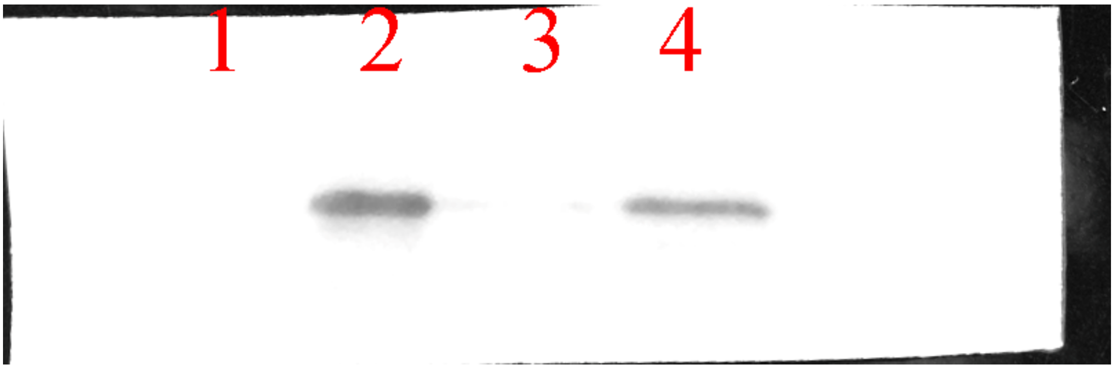


This figure shows the original data for Figure 6G. The order of lane loading from left to right was as follows: lane 1: Marker; lane 2: The supernatants of SUMO-Asf1. lane 3: The supernatants of GST and SUMO-Asf1 were passed through a GST gel column and then eluted with 10 mM GSH, which was detected by His antibody; lane 4: The supernatants of GST-B-like 3 and SUMO-Asf1 were passed through a GST gel column and then eluted with 10 mM GSH, which was detected by His antibody.

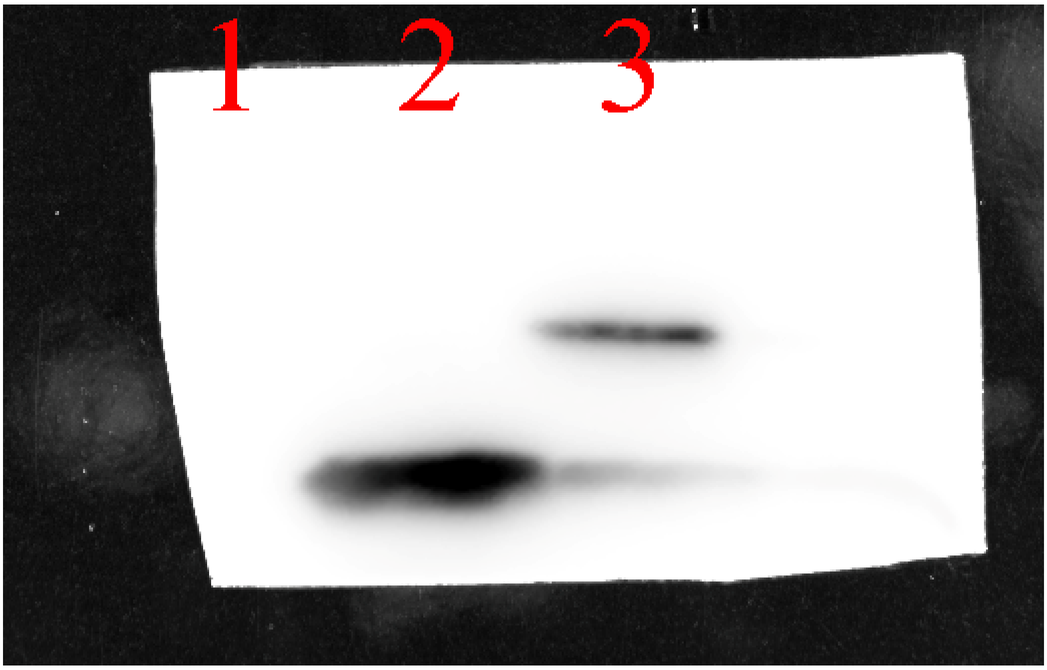


This figure shows the original data for Figure 6H. The order of lane loading from left to right was as follows: lane 1: Marker; lane 2: The supernatants of GST and SUMO-Asf1 were passed through a GST gel column and then eluted with 10 mM GSH, which was detected by GST antibody; lane 3: The supernatants of GST-B and SUMO-Asf1 were passed through a GST gel column and then eluted with 10 mM GSH, which was detected by GST antibody.

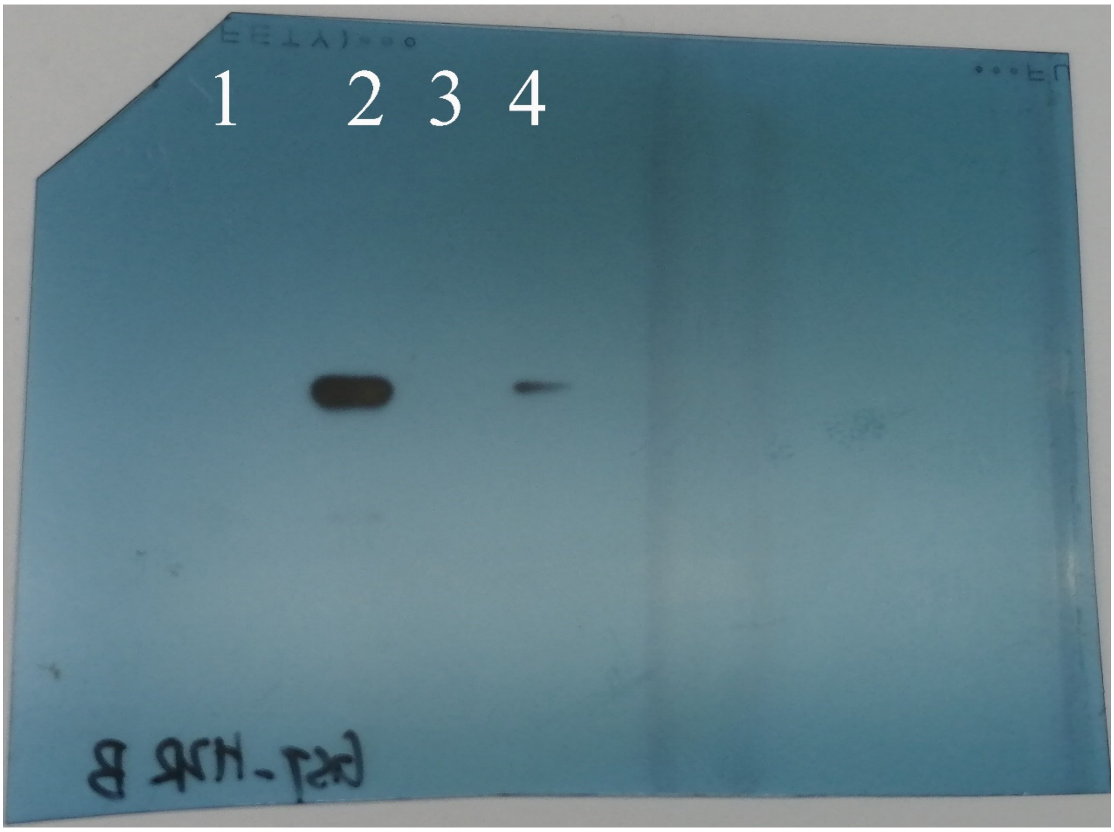


This figure shows the original data for Figure 6H. The order of lane loading from left to right was as follows: lane 1: Marker; lane 2: The supernatants of SUMO-Asf1. lane 3: The supernatants of

GST and SUMO-Asf1 were passed through a GST gel column and then eluted with 10 mM GSH, which was detected by His antibody; lane 4: The supernatants of GST-B and SUMO-Asf1 were passed through a GST gel column and then eluted with 10 mM GSH, which was detected by His antibody.

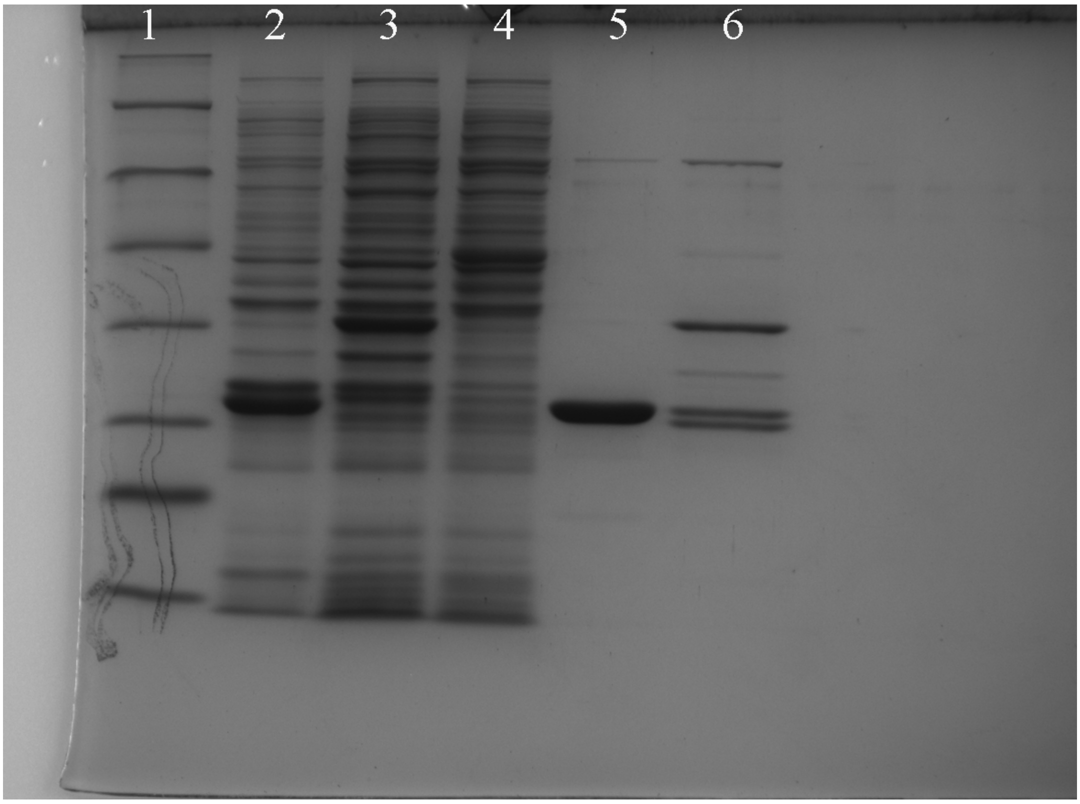


This figure shows the original data for Figure S7A. The order of lane loading from left to right was as follows: lane 1: Marker; lane 2: GST supernatant; lane 3: GST-B-like 1 supernatant; lane 4: SUMO-Asf1 supernatant; lane 5: The supernatants of GST and SUMO-Asf1 were passed through a GST gel column and then eluted with 10 mM GSH; lane 6: The supernatants of GST-B like 1 and SUMO-Asf1 were passed through a GST gel column and then eluted with 10 mM GSH.

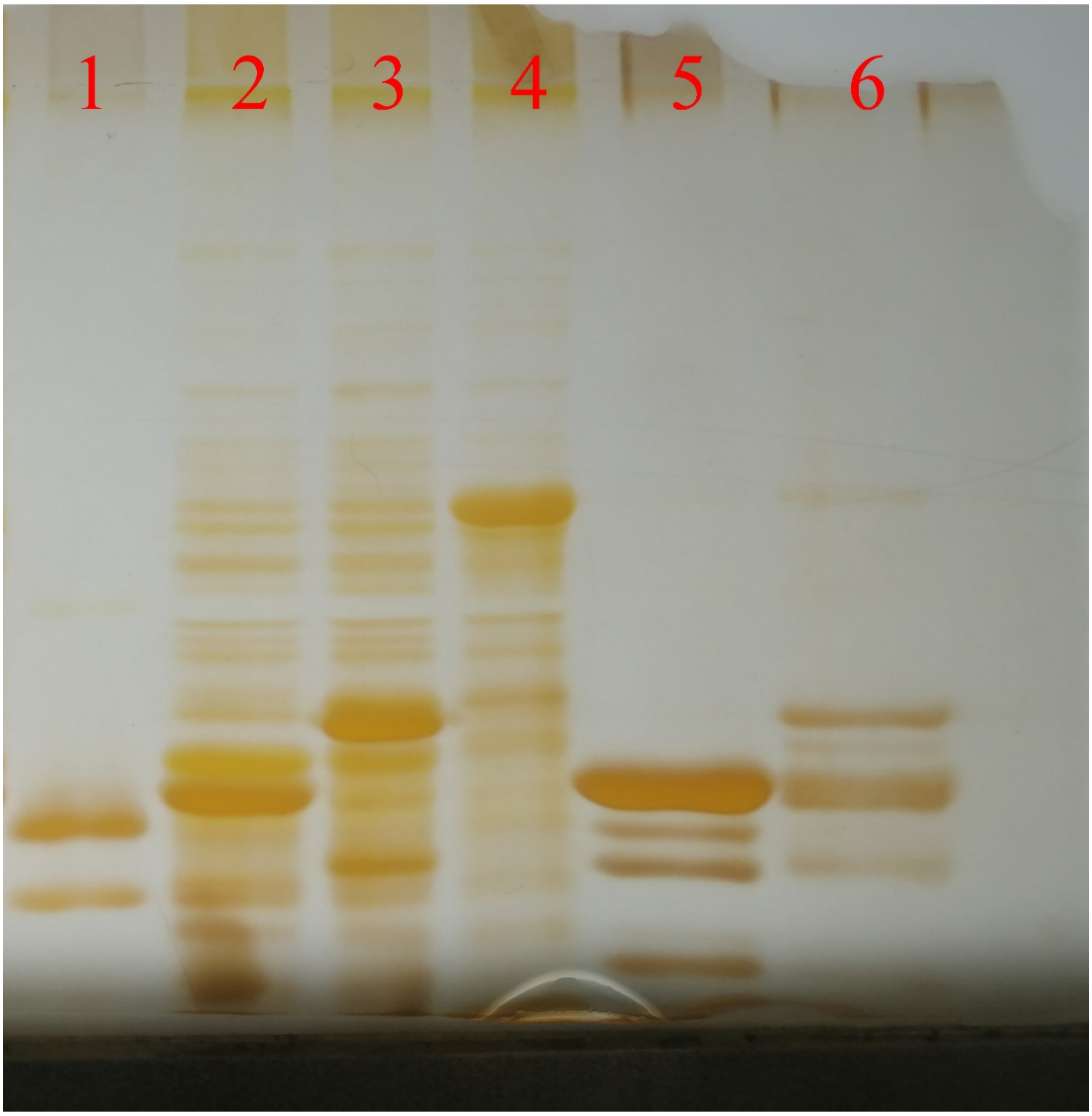


This figure shows the original data for Figure S7B. The order of lane loading from left to right was as follows: lane 1: Marker; lane 2: GST supernatant; lane 3: GST-B-like 2 supernatant; lane 4: SUMO-Asf1 supernatant; lane 5: The supernatants of GST and SUMO-Asf1 were passed through a GST gel column and then eluted with 10 mM GSH; lane 6: The supernatants of GST-B-like 2 and SUMO-Asf1 were passed through a GST gel column and then eluted with 10 mM GSH.

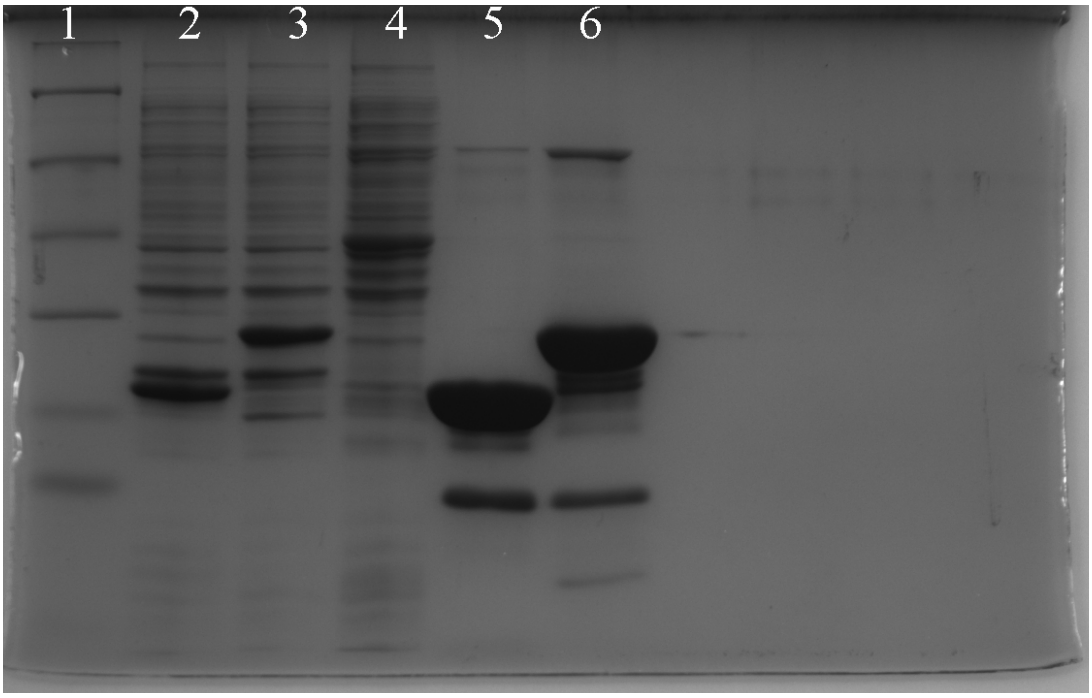


This figure shows the original data for Figure S7C. The order of lane loading from left to right was as follows: lane 1: Marker; lane 2: GST supernatant; lane 3: GST-B-like 3 supernatant; lane 4: SUMO-Asf1 supernatant; lane 5: The supernatants of GST and SUMO-Asf1 were passed through a GST gel column and then eluted with 10 mM GSH; lane 6: The supernatants of GST-B like 3 and SUMO-Asf1 were passed through a GST gel column and then eluted with 10 mM GSH.

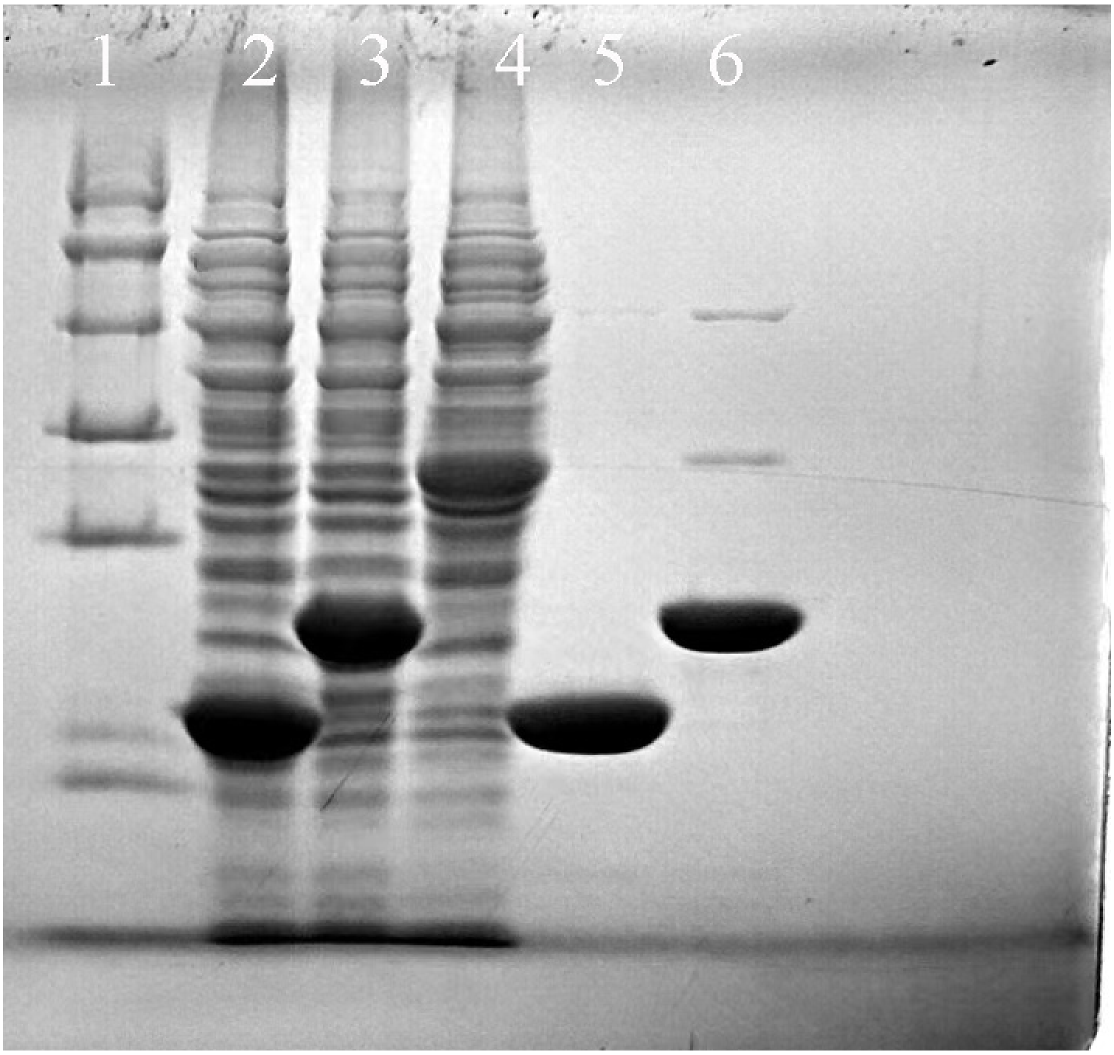


This figure shows the original data for Figure S7D. The order of lane loading from left to right was as follows: lane 1: Marker; lane 2: GST supernatant; lane 3: GST-B supernatant; lane 4: SUMO-Asf1 supernatant; lane 5: The supernatants of GST and SUMO-Asf1 were passed through a GST gel column and then eluted with 10 mM GSH; lane 6: The supernatants of GST-B and SUMO-Asf1 were passed through a GST gel column and then eluted with 10 mM GSH.

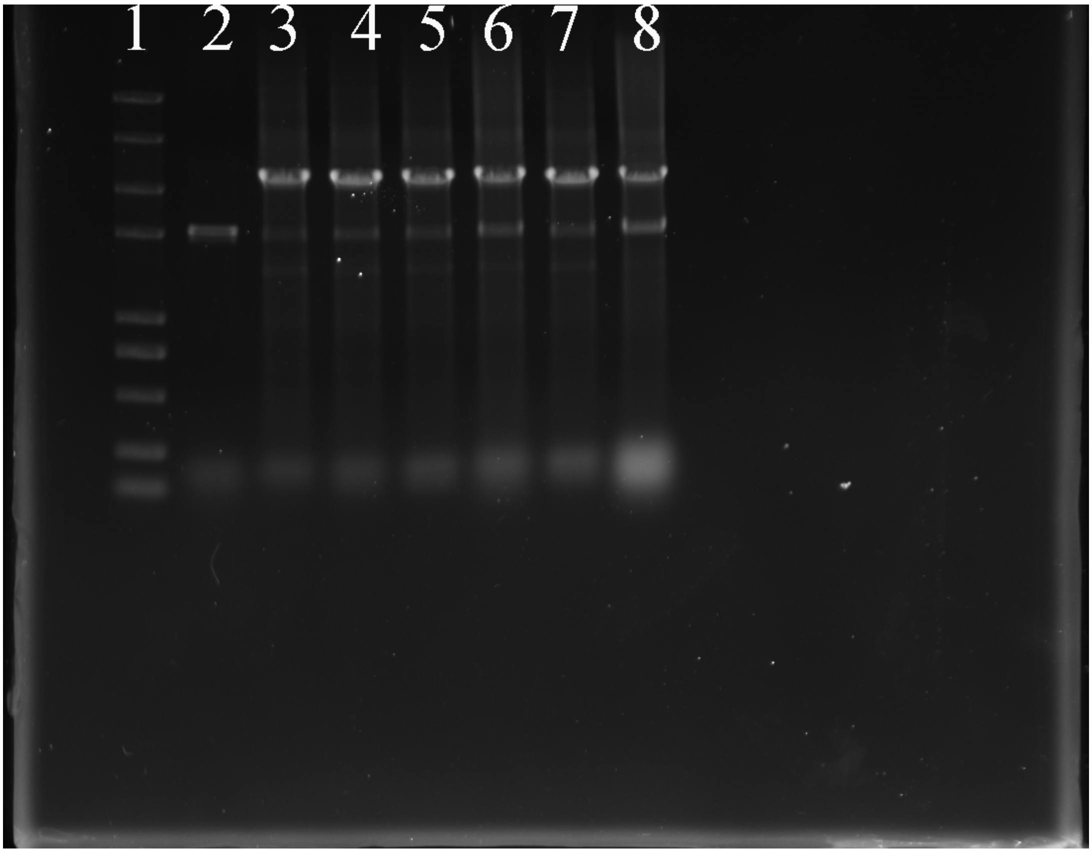


This figure shows the original data for Figure S2B. The order of lane loading from left to right was as follows: lane 1: Marker; lane 2: WT cells; lane 3-5: CAf1b-HA-B2086 mutant cell line; lane 6-8: CAf1b-HA-CU428 mutant cell line.

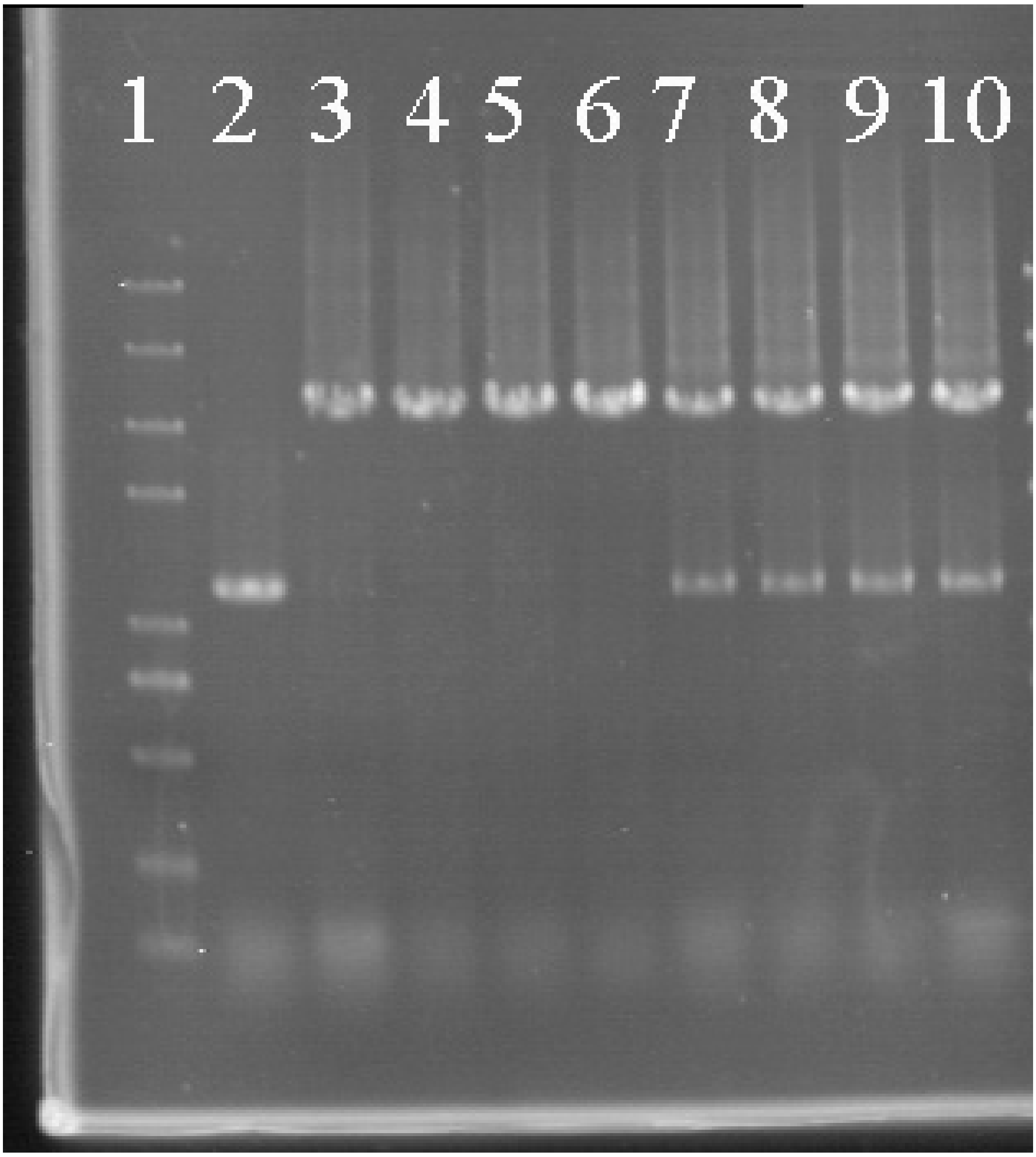


This figure shows the original data for Figure S2D. The order of lane loading from left to right was as follows: lane 1: Marker; lane 2: WT cells; lane 3-6: Hir1-HA-B2086 mutant cell line; lane 7-10: Hir1-HA-CU428 mutant cell line. Data after lane11 are not relevant to this article.

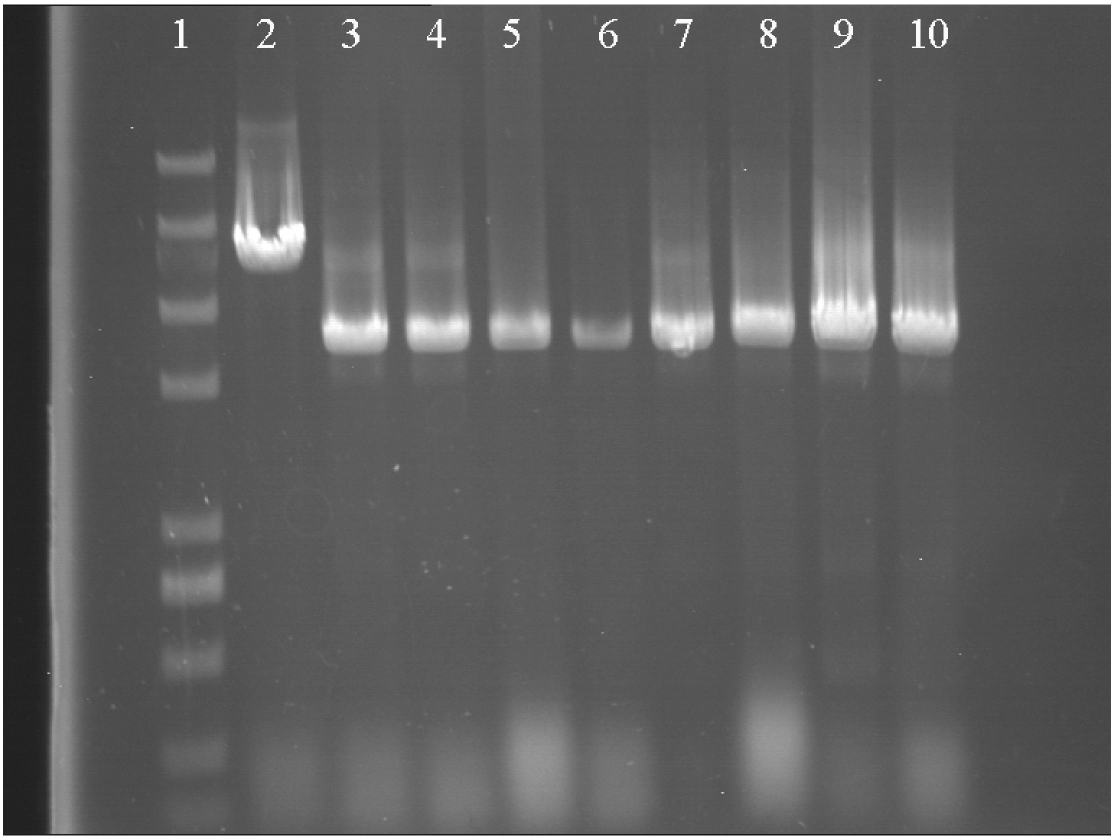


This figure shows the original data for Figure S4B. The order of lane loading from left to right was as follows: lane 1: Marker; lane 2: WT cells; lane 3-6: *HIR1*KD-B2086 mutant cell line; lane 7-10: *HIR1*KD-CU428 mutant cell line.

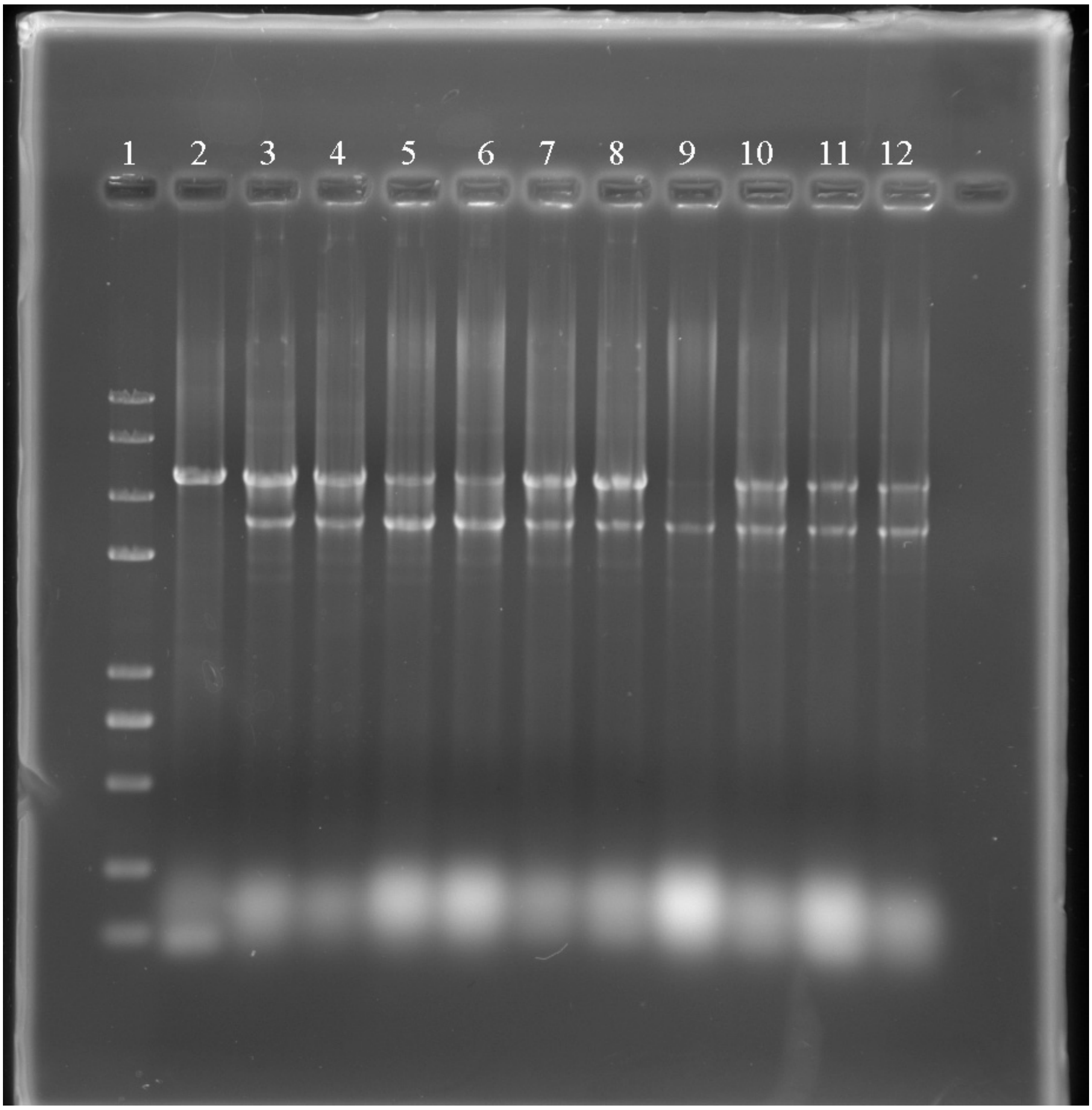


This figure shows the original data for Figure S5B. The order of lane loading from left to right was as follows: lane 1: Marker; lane 2: WT cells; lane 3-7: *CAF1B*KD-B2086 mutant cell line; lane 8-12: *CAF1B*KD-CU428 mutant cell line. Data after lane11 are not relevant to this article.

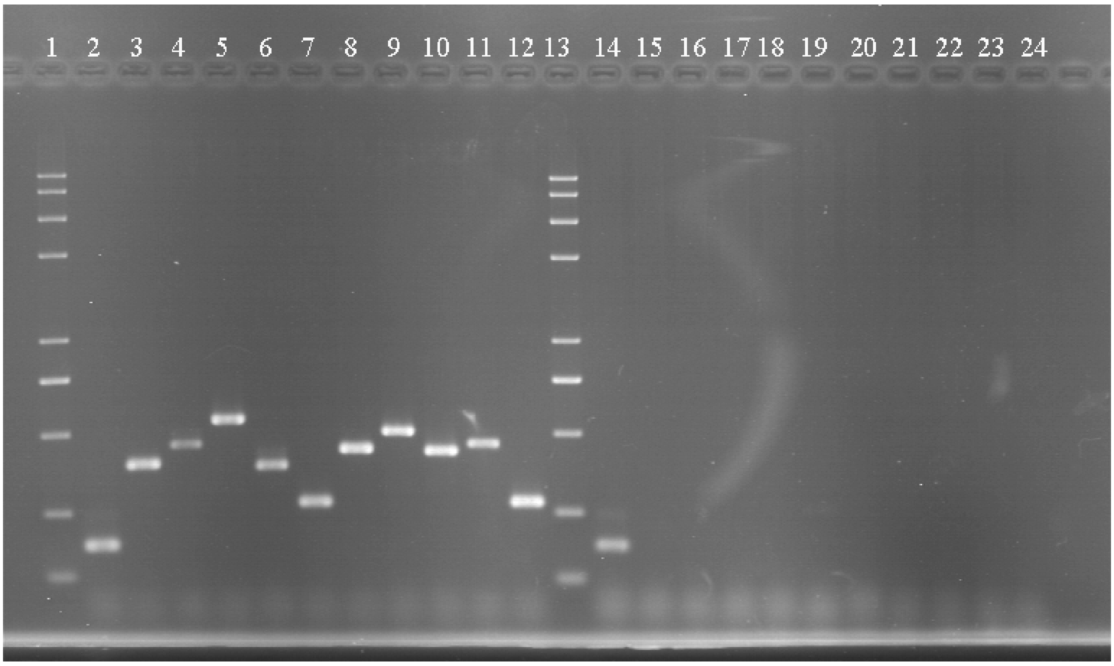


This figure shows the original data for Figure 4D. The order of lane loading from left to right was as follows: lane 1 and lane 13: Marker; lane 2 and line14: control; lane 3-12: Results of amplification of the wild-type genome with 10 pairs of MIC-specific primers; lane 15-24: Results of amplification of the *HIR1*KD genome with 10 pairs of MIC-specific primers.
